# Assessing the Spatial Distribution of Soil PAHs and their Relationship with Anthropogenic Activities at a National Scale

**DOI:** 10.3390/ijerph16244928

**Published:** 2019-12-05

**Authors:** Siyan Zeng, Jing Ma, Yanhua Ren, Gang-Jun Liu, Qi Zhang, Fu Chen

**Affiliations:** 1 School of Environment Science and Spatial Informatics, China University of Mining and Technology, Xuzhou 221008, China; syzeng@cumt.edu.cn (S.Z.); renyanhualucky@163.com (Y.R.); zhangqi2019@cumt.edu.cn (Q.Z.); 2 Low Carbon Energy Institute, China University of Mining and Technology, Xuzhou 221008, China; jingma2013@cumt.edu.cn; 3 Geospatial Sciences, College of Science, Engineering, and Health, RMIT University, Melbourne 3000, Australia; gang-jun.liu@rmit.edu.au

**Keywords:** polycyclic aromatic hydrocarbons, contamination, spatial variation, source distribution, soil quality, ecological safety

## Abstract

Soil polycyclic aromatic hydrocarbon (PAH) pollution is a major concern due to its negative impact on soil quality around the world. In China, accurate data on soil PAHs and information on the relationship with anthropogenic activities are limited. In this study, about 30,800 samples from 1833 soil sample sites were reviewed from 306 published reports to build a soil PAHs database. Based on the data obtained, the results demonstrated that 24.11% of surface soils in China are heavily contaminated. Meanwhile, the concentration of soil PAHs varied, in the order of independent mining and industrial areas (IMIA) > urban areas > suburban areas > rural areas, and the spatial distribution in China demonstrated a descending trend from north to south. Moreover, the characteristic ratio and PCA-MLR (principal component analysis-multiple linear regression) analysis demonstrated that coal combustion and vehicular exhaust emissions were the main sources of soil PAH pollution in China. On the other hand, provincial total Σ_16_PAHs in surface soil were significantly correlated with the per square kilometer GDP (gross domestic product) of industrial land, the per capita GDP, as well as the production and consumption of energy. These results indicate that anthropogenic factors have greatly affected the levels of soil PAHs in China. This study improves our understanding on the status and sources of soil PAH contamination in China, thereby facilitating the implementation of strategies of prevention, control, and remediation of soils.

## 1. Introduction

Polycyclic aromatic hydrocarbons (PAHs) are a group of persistent organic contaminants that are widely present in the environment. Some PAHs originate from natural sources, including volcanic eruptions and forest fires, among others. They also originate from emissions closely related to anthropogenic activities [1], such as incomplete combustion and pyrolysis of biomass and fossil fuels (i.e., crude oil, coke, petroleum, gas, and coal) [2,3]. Since most PAHs display a low water solubility, high octanol partition coefficient, and high binding capacity with organic matter, they are usually adsorbed into soil particles [4,5,6]. During the last two decades of rapid industrialization, soil media has become the most important sink for PAHs [7]. Because of their long-distance migration, refractory, and bioaccumulative qualities, PAHs have already become a hot spot in international society research, and the US Environmental Protection Agency (EPA) has issued a list of 16 PAHs [8] as priority pollutants. For this reason, their sources as well as concentrations, existing forms, behavior, biological effectiveness, and ecotoxicity require monitoring. The main global economies, including China, are paying significant attention to these chemicals [9,10,11,12].

Soil contamination with PAHs has been one of the most important environmental problems worldwide and requires an urgent solution [13,14,15]. At present, many places have been polluted with PAHs, such as Miami in the USA [16], Delhi in India [17], Beijing in China [18], Esbjerg in Denmark [19], Tyumen Oblast in Russia [20], and Ulsan in Korea [21,22], which, among others, have been studied by international researchers [23,24,25]. Nam et al. [26] found that the average background PAH concentration in different soils worldwide followed the order Europe > North America > Asia >Oceania > Africa > South America. Heywood et al. [27] used a spatial statistical analysis to demonstrate that in the British rural area, the western and southeastern sides are those with the highest PAH levels. Aichner et al. [28] studied 447 samples of German forest soils in order to determine which factors influence PAHs’ spatial distribution. Panagos et al. [29] illustrated that the contribution of municipal and industrial waste to soil contamination was as high as 38%, according to field contamination data collected by the European soil data center, and the PAH incidence was closely related to anthropogenic pollution. Jiao et al. [30] stated that large amounts of PAHs in the environment originate not only from the combustion of fuel and biomass but also from unexploited petroleum and coal. Soil is an important indicator of environmental pollution in PAHs, which can be a long-term reservoir of organic compounds [31,32,33]. The state of soil PAH pollution has received increasingly more attention in recent years [34,35,36].

In China, many predecessors have carried out significant research work on the status of soil PAHs pollution, finding that the pollution status and pollution sources in different regions have obvious differences. However, most of the investigations regarding PAH contamination have usually focused on small scales, including industrial and mining areas, garden green land, and wastewater irrigation sites [13,37,38,39]. At a national scale, researchers have usually adopted meta-analysis. For example, Cao et al. [40] analyzed 140 groups of PAH data points and reported that the level of contamination with PAHs from soils by point sources was significantly higher than contamination by non-point sources. Deng [41] collected data from references published between 2004 and 2007, and concluded that PAHs in surface soils in China mainly originate from the combustion of grass, wood, and coal. Ma et al. [42] found a large spatial distribution variation of PAHs in soils in China. For example, the highest Σ_16_PAH concentration was in urban soils, followed by rural areas and background soils. Applying the Maliszewska–Kordybach classification criteria [43], Zhang and Chen [44] determined that 82.8% of Chinese soils are contaminated with PAHs, where 21.4% can be classified as displaying high-risk pollution. Zhang et al. [45] used the bibliometrics method [46] to analyze data from 2000 to 2016, and found that the 16 PAHs requiring priority control in China surface soils were present in moderate levels as compared to those in other countries. Han et al. [47] reported that biomass, coal, and vehicle emissions should be classified as the main sources of PAHs in China. By using geographical detectors, Shang et al. [48] observed that the PAH content in surface soils in China was closely related to energy usage. Although previous studies have been very important for understanding PAH contamination in China, some information is still lacking, especially an in-depth analysis of the factors that affect the spatial distribution of PAHs and their regional accumulation, as well as an elucidation of the relationship between soil PAH pollution and anthropogenic activities at large scales.

The Chinese National Bulletin of Soil Pollution was issued in 2014. According to this publication, the excessive rate of soil PAHs in China was only 1.4% [49], which is totally different from previous estimations [42,44,47]. Recently, the consumption of energy generated from fossil fuels, such as petroleum and coal, has increased with increasing economic development and standards of living in China. This increased energy utilization has, in turn, caused PAH contamination in soil, water, and air. As a result, the environmental quality is deteriorating [50,51,52]. Although a significant number of reports regarding soil contamination with PAHs in China have been reported, some information is still lacking on PAH concentrations, such as clearer data on spatial distribution, especially about PAH accumulation and identification of sources and pollution processes using a systemic approach at a national scale. Furthermore, previous studies have not paid attention to the relationship between energy, economy, and urbanization activities. The 306 papers published by our predecessors measured the pollution levels of soil PAHs from different sites and accurately revealed the soil pollution in small areas of China. It is extremely important for us to understand the pollution status of PAHs in Chinese soil on a small scale, but it is difficult to explain the detailed characteristics of the contamination, accumulation processes, and spatial formation of PAHs at a national level. For this reason, the present study, based on 1833 soil sample sites with about 30,800 samples collected from 306 reports published from 2000 to 2019, systematically researched and investigated soil PAH contamination at a national scale. The specific purposes of this study were as follows: (1) To build a soil PAH concentration database for China; (2) to obtain soil PAHs’ spatial distribution; (3) and to determine the relationship between the provincial total Σ_16_PAHs in surface soil and anthropogenic activities.

## 2. Materials and Methods

### 2.1. Data Sources and Processing

**Figure 1:**
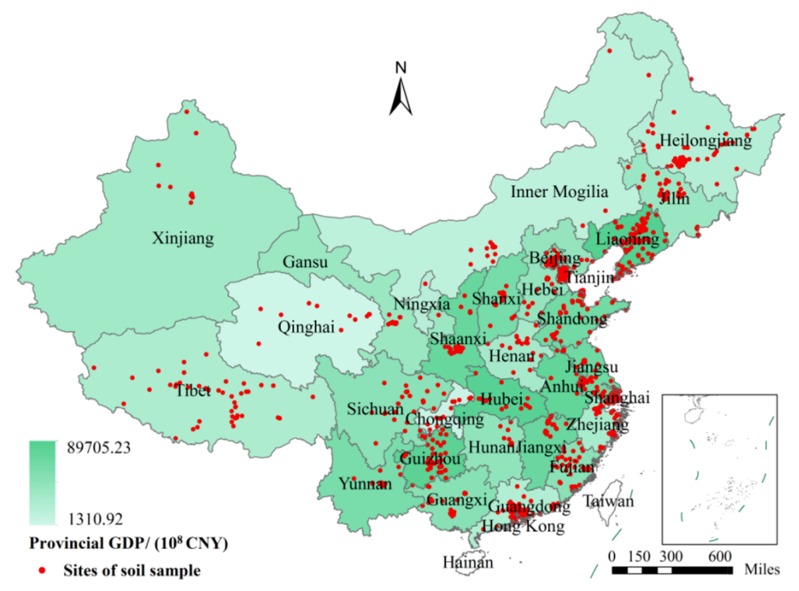
Spatial distribution of soil PAH (polycyclic aromatic hydrocarbon) sampling sites and provincial GDP (gross domestic product) of China in 2017.

In this work, the bibliometrics method [46] was used to collect related PAH data. Bibliographic references were obtained from the Web of Science and National Knowledge Infrastructure (CNKI) for the period 2000–2019 by applying the keywords “soil organic contamination” and “soil PAHs” to obtain 1833 soil sample sites (Figure 1). In order to select published reference data, the statistics included the investigated area, sampling point number, determination indexes, and clear determination results. Target data considered the surface soil at a depth between 0 and 40 cm. Graphic data or data directly given in the reference (single PAH or ΣPAHs) were included in the statistics, and data that could not be clarified or that only gave a numerical range were discarded.

In the present investigation, sampling point coordinates were confirmed by three methods: (1) The first one was by direct determination. The reference provided the detailed name and geographical coordinates of the sampling site as well as the contaminant type and content. (2) The second method was through name inversion to obtain geographic coordinates. In this case, Geosharp 2.1 (https://www.udparty.com, Shenzhen, China) was used to obtain the longitudinal and latitudinal coordinates of the sampling site. The systematic deviation of geographic coordinates in different references was removed by the combination of the transformation toolbox in the software. (3) The third method was a graphic digitalization of the sampling site. Data contained in figures showing the contaminant spatial distribution, and data in histograms and exponential graphs presenting the contaminant contents in the references were extracted using ArcGIS 10.1 (ESRI, Redlands, CA, USA) and Getdata 2.25 (http://www.getdatagraphdigitizer.com, GetData Pty Ltd., Moscow, Russia). Finally, data in 306 references reporting the PAH content in contaminated soils in China were evaluated. These data included records of 1833 sampling sites, about 30,800 sample points from all over China from 31 provinces and autonomous regions (Figure 1, Table S1).

**Figure 2:**
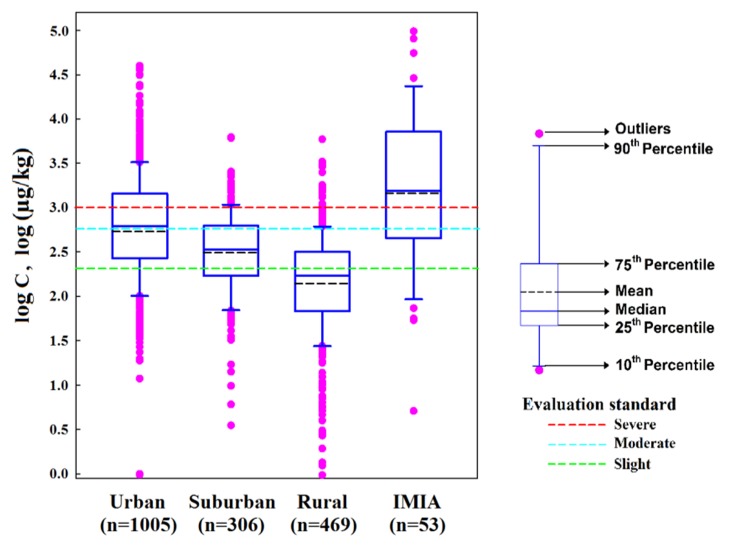
The log-transformed mean Σ_16_PAHs concentration (log C) in soil surface in rural, suburban, urban, and isolated mining and industrial areas (IMIA).

The box-shaped distribution diagram of soil PAH concentrations was plotted by Sigmaplot13.0, and consideration of soil utilization methods was used (Figure 2). A total of 1833 sampling sites were selected, and the distribution was as follows: 1005, 306, 469, and 53 samples corresponded to urban, suburban, rural, and independent mining and industrial areas (IMIA) areas, respectively. Moreover, the urban area was further divided into commercial (268), industrial (173), garden greenland (221), residential (63), traffic (164), cultural and educational (47), and other areas (69), where the figures in parentheses correspond to the number of sampling sites. PAHs included 16 individual compounds that were listed as contaminants with priority control in USEPA (the US Environmental Protection Agent) [53]. In addition, ΣPAHs refers to the original values of the Σ_16_PAH contents reported in each reference, not the arithmetic sum of the 16 individual PAHs compounds. Maliszewska–Kordybach [43] soil Σ_16_PAHs quality standards were used to classify the soils into four different pollution levels (clean corresponded to ≤200 µg/kg; slight, 200–600 µg/kg; moderate, 600–1000 µg/kg; and severe >1000 µg/kg). For sample data corresponding to the same type and collected in the same area, abnormal average concentration values in the range [x/4, 4x] were artificially removed [54]. These outliers are usually caused by anthropogenic activities, such as mining and industry, with great significance for small-scale research [55]. However, when large-scale spatial interpolation was performed, if these abnormal samples were not removed, areas with excessively high or low values would result in incorrect spatial interpolation.

Data on the amounts of energy reserves (petroleum, gas, and coal) in the Chinese provincial administrative areas and the production and consumption of coal and petroleum in 1997–2016 were obtained from the public service platform of Chinese energy data in 2016 (energy information association) (http://www.eia.org.cn). Information regarding the industrial land area in different provinces in 2017 was collected from the summary and analysis report of the Chinese urban land utilization data and national industrial land great survey data [56], which was issued by the National Land Resources Department of China. The industrial GDP and per capita GDP data for the year of 2017 were obtained from the “2018 China Statistical Yearbook” [57]. The value of the per square kilometer GDP of industrial land in each province was calculated by dividing the industrial GDP in 2017 by the industrial land area in different provinces (industrial GDP in 2017/provincial industrial land area, 100 million CNY/km^2^).

### 2.2. Statistical Analysis Method

Due to the large differences in the number of sampling points at each sample site, research with more soil PAH sampling points might make a greater contribution to the results of spatial interpolation at a regional or national scale [58,59]. The spatial distribution of the concentrations of PAHs in surface soil (100 m × 100 m raster data) at a national level was obtained by spatial interpolation, applying the inverse distance weighing (IDW) method with ArcGIS 10.1 (ESRI, Redlands, CA, USA). The cross-validating spatial distribution of PAHs in soils by using the selected soil samples from the new dataset included 130 sampling sites—about 2080 sample points from 31 reports (Table S2) [60].

The sample point numbers in each sample site were used as the weights when mapping the spatial distribution of PAHs in China. The sample-number-weighted means (*u*) of PAH concentrations were calculated with the following equation:

(1) $$u=\frac{B_{i}\times N_{i}}{\sum_{i=1}^{n}N_{i}},$$

where *N_i_* is the number of sampling points at sample site *i*, *B_i_* is the determined concentration of PAHs at sample site *i* obtained from 306 peer-reviewed articles, and *n* is the number of sample sites:

(2) $$µ\left(x\right)=\{\begin{array}{c} \frac{\sum_{i=1}^{N}w_{i}\left(x\right){µ}_{i}}{\sum_{i=1}^{N}w_{i}\left(x\right)},\;d\left(x,\;x_{i}\right)\neq 0\\ u_{i},\;d\left(x,\;x_{i}\right)=0\; \end{array},$$

(3) $$w_{i}\left(x\right)=\frac{1}{d{\left(x,x_{i}\right)}^{r}},$$

where *w_i_* (*x*) is the interpolation weight function. For an interpolated value, *µ*, at a given point, *x*, based on samples *µ_i_* = *µ* (*x_i_*), for *i* = 1, 2, …, N is the number of known points, *x_i_*, using the interpolation; *d* is the distance from point *x_i_* to *x*; and *r* is the weight coefficient:

(4) $$A_{m}=\sum µ\left(x\right)r,$$

where *A_m_* is the total Σ_16_PAHs in the surface soil of province m, *µ*(*x*) is the sample-number-weighted mean of the PAH concentration by applying the inverse distance weighing (IDW) method, and r is the number of raster grids in province m. By using the regional analysis module of the Spatial Analyst tool with ArcGIS 10.1, the provincial administrative areas of China were selected as regional vector data, and factor extraction was performed in units of provinces. The statistical type was selected as “sum”, and the regional statistics of the total amount of provincial polycyclic aromatic hydrocarbons (provincial total Σ_16_PAHs in surface soil) were calculated.

Different source apportionment and receptor-oriented approaches have been developed and applied to identify the sources of PAHs in contaminated soil [61], such as the characteristic ratio method, positive matrix factorization (PMF), principal component analysis-multiple linear regression (PCA-MLR), unmix model, etc. [62,63]. Considering the differences in the composition and proportion of PAHs between different sources of pollution, the characteristic ratio method (considering the ratio of isomers with the same molecular weight) was used to identify the source of PAHs in this study. However, there would be some limitations if we just used the PAH diagnostic ratio method, because the source of contamination and the ratios could be modified in environmental processes, such as oxidation and photo-oxidation [64]. Moreover, the use of multiple techniques is generally recommended to minimize the bias of the individual method and increase the reliability of the source diagnosis results [65]. Therefore, the characteristic ratio method and PCA-MLR method were combined to determine the source distribution of soil PAH pollution in this study. PCA-MLR is a quantitative method that is used to determine the emission proportion by extracting the principal components with eigenvalues greater than 1, performing regression analysis on the factor analysis results, and calculating the contributions of different pollution sources [66,67]. Furthermore, considering the main source differences between LMW (low-molecular weight PAHs, 2–3 rings) and HMW (high-molecular weight PAHs, ≥4 rings), the Pearson correlation coefficient (SPSS 19.0, IBM, Armonk, NY, USA) was used to analyze the energy indicators (independent variable) and provincial PAH content (dependent variable), revealing the main factors influencing regional PAH accumulation [68,69].

## 3. Results

### 3.1. Characteristics and Spatial Distribution of PAHs in Soils in China

According to the data analysis of the statistical references used in the present study, the Σ_16_PAHs concentration in surface soils in China is between 0.02 and 97,680.00 µg/kg (CV = 3.47), with a relatively large regional variation. The highest PAH concentration was found in the Liaohe oil field [70]. The national arithmetic mean concentration was of 1209.35 µg PAHs/kg. Based on the soil Σ_16_PAHs quality standards provided by the Maliszewska–Kordybach classification criteria, 70.65% of soils in China tested in this study displayed PAH values above the standard rate, mainly at the level of weak contamination. Specific data were 33.39%, 13.15%, and 24.11% corresponding to weak, moderate, and heavy contamination, respectively. In addition, the non-contamination proportion was only 29.35%. Considering the arithmetic mean of the PAH content in the surface soils of the 1833 sampling sites included in the present investigation, the following descending order was observed: IMIA > urban area > suburban area > rural area, corresponding to concentrations of 8800.70 > 1479.97 > 523.34 > 293.95 µg/kg, respectively (Figure 2).

**Figure 3:**
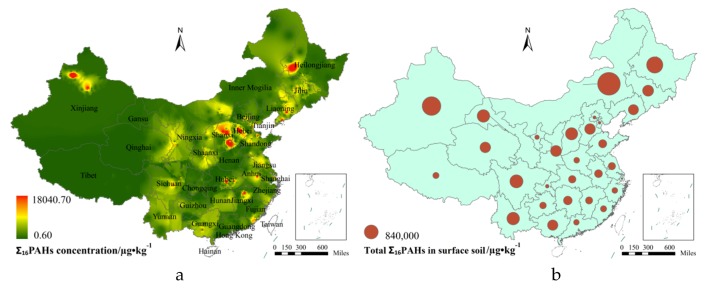
Spatial distribution of Σ_16_PAHs (**a**) and provincial total Σ_16_PAHs in surface soil (**b**).

According to the spatial interpolation results (Figure 3a), the spatial distribution of PAHs in soils displayed a characteristic aggregation, with multiple centers with areas of high concentrations in the northwest old industrial base (the west of Heilongjiang, the middle of Jilin and Liaoning), the north of Shandong, the middle of Shanxi, and the south of Hebei. Also, the Beijing-Tianjin-Tangshan region, Sichuan, the middle of Jiangxi, Anhui, and the south of Fujian had a small number of areas with high concentrations. No significant high values were found in metropolitan areas in China, such as the Yangtze River Delta and the Pearl River Delta. Data analysis of four economically developed areas in China (northeast old industrial base, Beijing-Tianjin-Tangshan region, Yangtze River Delta, and Pearl River Delta) indicated a descending order for the average concentration of PAHs in soils as follows: Northeast old industrial base (1800.43 µg/kg) > Beijing-Tianjin-Tangshan region (1762.32 µg/kg) > Yangtze River Delta (995.51 µg/kg) > Pearl River Delta (394.52 µg/kg).

The spatial distribution of provincial total Σ_16_PAHs in the surface soil of China was obtained by considering each province as a statistical unit and applying the regional analysis module (Spatial Analyst) (Figure 3b). The results indicate that the spatial distribution in China has a descending trend from north to south, both for PAH concentrations and provincial total Σ_16_PAHs in surface soil, and concentrations in the middle, south, and southeast areas are relatively lower. The three provinces with the highest provincial total Σ_16_PAHs in surface soil were found to be Inner Mongolia (2,209,243.50 µg/kg), Xinjiang (1,498,492.00 µg/kg), and Heilongjiang (1,136,955.88 µg/kg).

### 3.2. Determination of the Source of PAHs in Soils via Multiple Approaches

**Figure 4:**
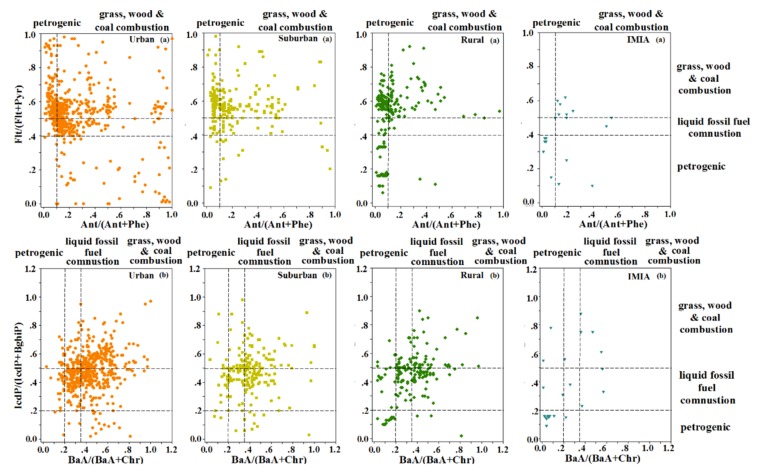
Isomeric ratios obtained by plotting Ant/(Ant + Phe) vs. Flt/(Flt + Pyr) (**a**) and BaA/(BaA + Chr) vs. IcdP/(IcdP + BghiP) (**b**).

According to previous reports [71,72], a Flt/(Flt + Pyr) (fluoranthene/(fluoranthene + pyrene) value below 0.4 is characteristic of a petroleum source. In addition, if this figure is between 0.4 and 0.5, this corresponds to the combustion of petroleum-like substances. When the resulting value is larger than 0.5, it is very likely that the PAHs originated from the combustion of coal and wood. The boundary value of the Ant/(Ant + Phe) (anthracene/(anthracene + phenanthrene) ratio was 0.1; namely, values below and above 0.1 corresponded to petroleum and combustion sources, respectively. The IcdP/(IcdP + BghiP) (indeno (1,2,3-cd) pyrene/(indeno(1,2,3-cd) pyrene + benzo (g,h,i) perylene) ratio below 0.2 indicated petroleum contamination, ratio values between 0.2 and 0.5 implied the combustion of petroleum-like substances, and those values above 0.5 indicated the combustion of coal and wood. The BaA/(BaA + Chr) (benza (a) anthracene/(benza (a) anthracene + chrysene) ratio below 0.2 implied a petroleum source; from 0.2 to 0.35 indicated the combustion of petroleum and fossil fuel, and values above 0.35 the combustion of biomass and coal. The results in Figure 4 show that all PAH pollution sources were reflected in the surface soil of China, which indicates that some deviations might occur when a PAH source analysis is conducted using only the ratio method at a national scale.

**Figure 5:**
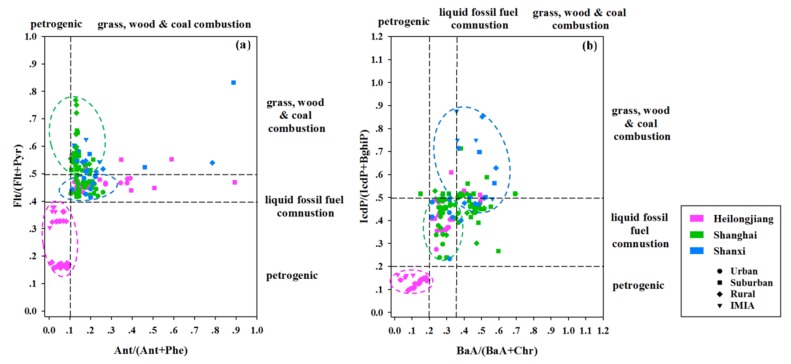
Isomeric ratios obtained by plotting Ant/(An t+ Phe) vs. Flt/(Flt + Pyr) (**a**) and BaA/(BaA + Chr) vs. IcdP/(IcdP + BghiP) (**b**) in Heilongjiang, Shanghai, and Shanxi.

However, when using the characteristic ratio method to judge the source of soil PAHs, significant differences were found between the three special provinces of Heilongjiang, Shanghai, and Shanxi (Figure 5). For Heilongjiang (typical petroleum exploitation area), suburban and IMIA areas were greatly affected by the activity of petroleum exploitation, and soil PAHs were derived from petroleum contamination. For Shanghai (characteristic energy consumption area), most of the collected soil samples, including urban, suburban, and rural areas, were mainly derived from the combustion of petroleum-like substances, coal, and wood. However, for Shanxi (traditional coal mining area), except for urban samples, rural, suburban, and IMIA areas are mainly affected by coal mining, and most of these areas come from coal and biomass burning sources.

**Figure 6:**
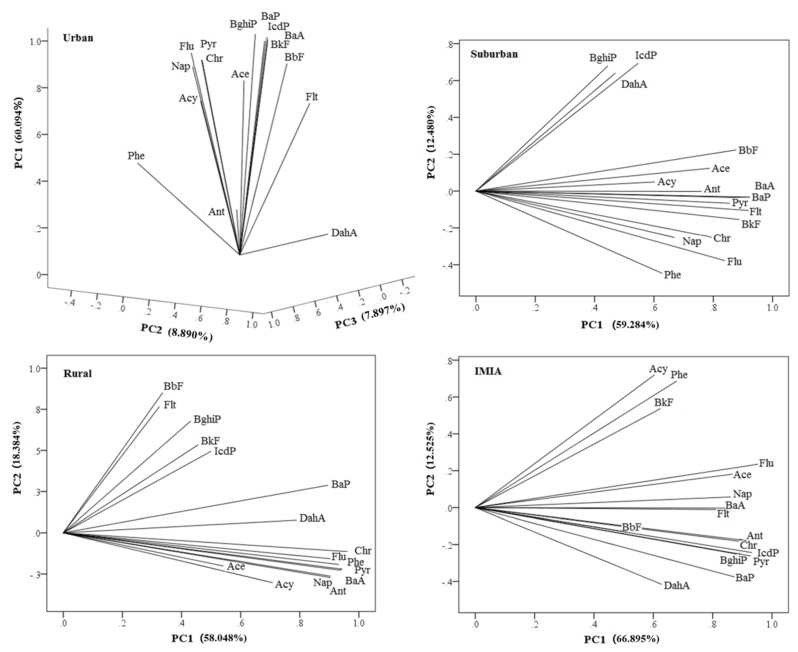
Principal component analysis of soil PAHs for urban, suburban, rural and IMIA areas.

Because of the simple use of the ratio method, deviation may exist, so the simultaneous PCA-MLR was used to properly identify PAH sources for further judgment. Figure 6 presents the results of PAHs in surface soils in urban, suburban, rural, and IMIA areas. Three factors related to urban soil PAH sources were extracted. Factor 1 explained 60.094% of the variability. Usually, Pyr, BaA, Chr, BbF, BkF, and BaP are indicators of coal combustion [7]. Thus, the factor 1 represented coal combustion as the PAH source. The factor 2 loading of DahA, which represented the specific indicator of vehicular exhaust contamination, was higher [73]. The loading of factor 3 was 7.897%, where the loading of Phe was higher, representing the source of biomass combustion [71]. Suburban soil PAH sources extracted two primary factors. Factor 1 represented sources of coal combustion, explaining 59.284% of the total variability, and factor 2 explained 12.480%. For factor 1, the loading coefficients for PAHs with four to five rings (Flt, Pyr, BaA, Chr, BbF, BkF, and BaP) were significantly high. Furthermore, the loading coefficient of Flu was also very high. Related research regarded Flu as one of the characteristic compounds in the process of coke production from coal [72]. IcdP, which represented the characteristic indicator of vehicular exhaust contamination, displayed the highest loading coefficient [74]. In rural soils, two factors were extracted for PAH sources. In relation to factor 1, compounds characteristic of coal combustion (BaA, Chr, BkF, BaP) and low-molecular weight PAHs (two to three rings) are highly volatile. Phe and Ant represented material generated in the incomplete combustion of straw and wood. Factor 2 explained 18.384% of the total variability. Compounds with high loading coefficients were Flt and BbF, which were compounds characteristic of coal combustion. Two factors were extracted for PAH sources of the IMIA sites, explaining 66.895% and 12.525% of the total variability, respectively. Factor 1 compounds with high loading coefficients were the main products of coal combustion (Flt, Pyr, BaA, Chr, BaP) and vehicular exhaust (IcdP, BghiP). The highest load factor of factor 2 was Acy, which was the main product from a petroleum source.

The results of the variance contribution rate (%) were obtained by using principal component analysis of soil PAHs for urban, suburban, and rural areas and IMIA. Then, the regression analysis of the above factor analysis results was performed to reflect the contribution of each major source (contribution of PAHs sources (%)) by using the linear regression method model. The results of the contributions of different pollution sources show that coal combustion (65.639%), automobile exhaust emissions (17.747%), and biomass (16.614%) are the main sources in urban soil. For suburban soil, coal combustion and automobile exhaust emissions accounted for 76.831% and 23.169%, respectively. The sources of PAHs in rural soil are mainly from coal combustion, with a contribution rate of 91.002%, followed by biomass, accounting for 8.998%, and the sources of PAHs in IMIA areas are mainly coal and petroleum combustion, and the contribution of petroleum combustion sources was as high as 34.157%. This confirms this activity as the main energy structure in China. In addition, vehicular exhaust emission was also an important contribution to this type of pollution in China. It has been reported that with increasing living standards, the number of residential vehicles has also exceeded 100 million in 2012 and reached 209.6 million in 2017 [57]. As consequence, vehicular exhaust emissions have also been augmented. In relation to rural soil, the main PAH sources were the combustion of coal and straw and wood biomass. Analysis of the collected data indicated that fractions of Phe (a compound representative of the incomplete combustion of straw and wood) [71] were present in the following descending order: Rural area (16.30%) >suburban area (14.13%) >urban area (12.00%). Except for urban soils, the main sources of PAHs in the other three areas were coal combustion, and the contribution rate of petroleum leak (PC2) in the IMIA area was as high as 34.157%. These two were related to changes in energy structure, namely the gradual decrease in coal consumption, as well as the gradual increase in oil utilization. Companies with high energy consumption, especially those dedicated to petroleum refining processes, coke plants, and iron and steel production, are usually more distributed in suburban, rural, and IMIA areas. All of them possibly contribute to the accumulation of PAHs in soils.

### 3.3. Relationship between PAHs Contamination and Anthropogenic Activities

PAHs concentrations in surface soils in IMIA and urban areas in China were far higher than those observed in suburban and rural areas. The spatial interpolation results (Figure 3a) showed that areas with high soil PAH concentrations were those related to petroleum and chemical engineering activities. For example, the Daqing oil field in the west of Heilongjiang, the Shenli oil field in the north of Shandong, the Jianghan oil field in the middle of Hubei, the Huaibei oil field in the south of Hebei, the Kelamayi oil field in the north of Xinjiang, and the Dagang oil field in the south of Tianjin [70,75,76]. Another high contributor to PAHs was the exploitation of coal deposits and the generation of thermal power, which was observed for Shanxi and Xuzhou cities in the north of Jiangsu [77,78]. The third important contribution was the developed industries in the urban area, e.g., Tiexi in Liaoning, Dagang in Tianjin, and Lanzhou in Gansu [79,80,81].

According to the information obtained from the public service platform of China’s energy data (energy information association) (http://www.eia.org.cn/), the highest base reserves of the three main sources of energy (petroleum, gas, and coal) in different areas of China in 2016 were Xinjiang (595,763.0 thousand tons), Sichuan (1319.161 billion cubic meters), and Shanxi (91.619 billion tons). Results from the Pearson correlation analysis indicated that concentrations of LMW PAHs in soils were positively correlated with petroleum reserves (*r* = 0.695, *p* < 0.01). In addition, the content of HMW PAHs was positively correlated with gas reserves (*r* = 0.571, *p* < 0.01), coal reserves (*r* = 0.655, *p* < 0.01), coal production (*r* = 0.677, *p* < 0.01), and total crude oil and coal production and consumption (*r* = 0.565, *p* < 0.01) (Table 1). Usually, LMW PAHs mainly originate from petroleum contamination while HMW PAHs are mainly from incomplete coal combustion at high temperatures [82,83]. The three Chinese provinces with the highest provincial total Σ_16_PAHs in surface soil were Inner Mongolia (the province with high coal combustion activity), Xinjiang (the main concentration of petroleum and gas reserves in China), and Heilongjiang (old industrial base). The provincial total Σ_16_PAHs in surface soil was positively correlated with petroleum reserves (*r* = 0.476, *p* < 0.05), gas reserves (*r* = 0.643, *p* < 0.01), coal reserves (*r* = 0.491, *p* < 0.01), coal production (*r* = 0.540, *p* < 0.01), as well as total crude oil and coal production and consumption (*r* = 0.492, *p* < 0.01). These results further confirmed that PAH pollution in these areas is caused by natural sources and anthropogenic factors, such as coal combustion and petroleum.

**Figure 7:**
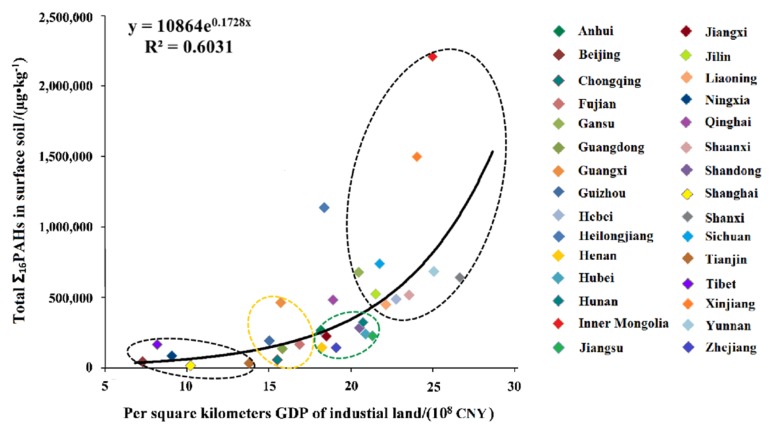
The relationship between the per square kilometer GDP of industrial land in 2017 and provincial total Σ_16_PAHs in the surface soil of China.

Based on China’s industrial land survey data [56] and the 2018 China Statistical Yearbook [57], the value of per square kilometer GDP of industrial land in each province (industrial GDP in 2017/provincial industrial land area, 100 million CNY/km^2^) was calculated. In addition, a correlation analysis between the provincial total Σ_16_PAHs in surface soil and per square kilometer GDP of industrial land was conducted (Figure 7). The results demonstrated a positive correlation between the provincial total Σ_16_PAHs in surface soil and the per square kilometer GDP of industrial land (R^2^ = 0.6031). The per square kilometer GDP of industrial land and provincial total Σ_16_PAHs in the surface soil of Beijing, Tibet, Ningxia, Shanghai, and Tianjin were lower than those in other provinces in China. However, the values of these two indicators were found to be relatively high in the 10 provinces of China, such as Inner Mongolia, Xinjiang, Sichuan, Yunnan, and Shanxi, etc. The differences between the per square kilometer GDP of industrial land and provincial total Σ_16_PAHs in the surface soil of Chongqing, Guizhou, Guangxi, Guangdong, and Fujian provinces were small, showing a concentrated distribution; these five provinces mainly fall in southwestern and southern China. In addition, an aggregated distribution was observed in those eight provinces (Jiangxi, Hunan, Hubei, Henan, Zhejiang, Anhui, Jiangsu, and Shandong), which were located in central and eastern China. The lowest provincial total Σ_16_PAHs in surface soil was located in Shanghai, and the highest was found in Inner Mongolia. A descending trend for provincial total Σ_16_PAHs in the surface soil of the three provinces in northeast China was obtained as follows: Heilongjiang (1,136,955.88 µg/kg) > Jilin (524,345.06 µg/kg) > Liaoning (449,303.75 µg/kg). This may be a result of the petroleum exploitation activities in the Daqing oil field in the Heilongjiang province.

## 4. Discussion

### 4.1. Status of Surface Soil Contamination with PAHs in China

Most of the previous research reporting soil contamination with PAHs in China has focused on small areas. In this work, we collected data from a great number of references, which were evaluated by applying the Maliszewska–Kordybach classification criteria. Results indicated that from the total collected data, 70.65% surpassed the standard rates, with 33.39%, 13.15%, and 24.11% corresponding to weak, moderate, and heavy contamination, respectively. Our weak contamination ratio results were similar to those obtained by Cai et al. (34%) [84] and Deng et al. (31%) [41]. However, it was far lower than the value published by Zhang and Chen (49.5%) [44]. Moderate and heavy contamination ratio results were comparable to values reported by Zhang and Chen (11.9% and 21.4%, respectively) [44]. According to our data, 29.35% of the soil samples contained no PAH contamination; this figure is higher than those of Cai et al. (22%) [84], Deng et al. (23%) [41], Zhang and Chen (17.2%) [44], and Shang et al. (6.3%) [48]. A possible reason for this difference may be the relatively comprehensive sample database used in the present work. Moreover, when the Σ_16_PAH concentration in rural areas was far lower than those identified in other zones, the representative phenanthrene (Phe) fraction still displayed higher values than those observed in suburban and urban sites. As we know that Phe is always generated through the incomplete combustion of straw and wood in rural areas, the result might be greatly related to the input of PAHs in rural areas [85,86,87].

Dominant industries in IMIA are the long-term exploitation and processing of mineral resources, such as petroleum and coal. There are many petroleum-processing companies, electric power plants, and steel and iron plants in suburban areas, whereas the urban area displayed a high development level, a large traffic flow, and advanced traffic networks. These conditions favor the input, accumulation, and distribution of PAHs in surface soils in China.

In general, compared with other regions of the world, the concentration of PAHs in soils in China was present at a moderate level (Table 2). The average PAH concentration in urban soils was lower than those reported for Delaware in the USA (2732 µg/kg) [88], Delhi in India (5524.25 µg/kg) [89], Bursa in Turkey (2243.5 µg/kg) [90], and London in the UK (68,000 µg/kg) [91]. On the other hand, the value obtained in the present research was dramatically higher than that of Korea (390 µg/kg) [21]. Suburban and rural soil PAHs concentrations in China were all lower than the reported values for Delhi in India [92] and England [93], and higher than those of Delaware in the USA [88], South Korea [94], and Bursa in Turkey [90]. PAH levels in the studied IMIA soils were 3.50-, 2.25-, and 1.90-fold higher than those reported for Ulasn in Korea [95], Bajalkota in India [96], and Izmir in Turkey [97], respectively. It is worth noting that the maximum concentrations of PAHs in urban, rural, and IMIA soils were 3.48, 2.82, and 7.69 times that of the concentration in Bajalkota in India [96]. PAHs in soils usually display a natural background concentration of 1 to 10 µg/kg, and they mainly originate from plant decomposition and natural fires [98]. Concentrations of PAHs far higher than natural values indicate that the soils have been influenced by anthropogenic factors, including atmospheric dry and wet deposition, chemical inputs, agricultural wastewater and soil, coal combustion, petroleum consumption, and accidental leaks, among others [99]. In this work, only 2.84% of the total number of sampling sites (1833) presented concentrations lower than the natural background value (<10 µg/kg). This may indicate that most soils in China have been influenced by anthropogenic activities. Even when the level of soil pollution by PAHs in China can be classified as moderate as compared with other countries, attention should be paid to those areas with high contamination levels, such as the northeast old industrial base and those with chemical engineering- and petroleum-related activities.

### 4.2. Spatial Distribution of Soil PAHs Hot Spot Areas in China

In this study, the spatial distribution in China displayed a descending trend from north to south both for the PAH concentrations and provincial total Σ_16_PAHs in surface soil, and those in the middle, south, and southeast areas were relatively lower. The results were similar to those issued by Cao et al. [40] and Zhang and Chen [44], who determined that PAH concentrations in surface soils in this country occurred in descending order from northern to southern China by collecting data from 1999 to 2008 and from 2006 to 2016, respectively. Due to the low temperatures in the northern areas, the evaporation and diffusion of PAHs is limited [100]. This might be one of the reasons for the presence of high levels of PAHs in surface soils in the northern areas of China. On the other hand, some PAHs show volatile properties. High temperatures and sufficient light in southern areas might facilitate the evaporation and diffusion of PAHs, regardless of whether they are generated from contamination sources or are naturally occurring. As a result, concentrations of PAHs in the southern area were relatively lower than in the northern area.

In this work, the PAH contamination concentration data of soil samples were collected using the bibliometrics method [46]. The land use of collected sampling sites was mainly industrial and commercial. Spatial hot spots were predominantly located at the northeast old industrial base, Beijing-Tianjin-Tangshan region, Yangtze River Delta, and Pearl River Delta. The average PAH concentrations in soils of the four spatial hot spot areas were, in descending order, northeast old industrial base > Beijing-Tianjin-Tangshan region > Yangtze River Delta > Pearl River Delta. These data are in accordance with the reports of Deng et al. [41], demonstrating that the average soil PAH concentrations for a specific area in China might decrease with decreasing altitude [40]. In addition, the decreasing trend of PAH concentrations from north to south might be influenced by climate change. The soil PAH concentrations in the northeast old industrial base and the Beijing-Tianjin-Tangshan region were 1.80- and 1.77-fold higher than that of the Yangtze River Delta, respectively. They were also 4.56- and 4.46-fold higher than the values obtained for the Pearl River Delta. These results demonstrate that energy production and consumption might also affect the accumulation of soil PAHs, except for economic development.

### 4.3. Anthropogenic Factors Influencing the Presence of Soil PAHs in China

**Figure 8:**
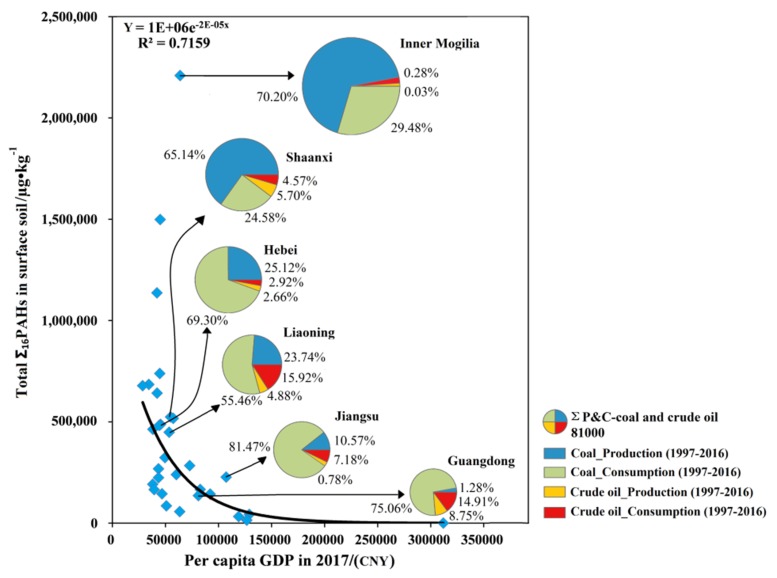
The relationship between per capita GDP and provincial total Σ_16_PAHs in the surface soil of China.

Anthropogenic emissions are an important source of PAHs in the environmental media [101,102,103]. Li et al. [104] pointed out that the accumulation of Σ_16_PAHs in Chinese lake sediments has rapidly increased since the 1980s, which is highly related to the rapidly increasing social economic development level and the total energy consumption. This study demonstrated a positive correlation between the provincial total Σ_16_PAHs in surface soil and the per square kilometer GDP of industrial land (R^2^ = 0.6031) (Figure 7). It was also shown that the level of industrial development was the important factor, which influenced the total concentration of PAHs in soils in China. The level of industrial development was directly related to fossil fuel consumption, and emissions of industrial wastewater and garbage, among others. Thus, it influenced the level of PAH emissions in the environment [105]. In particular, resource exploitation activities, such as crude oil and coal, have obvious effects on soil PAH pollution in suburban, rural, and IMIA areas. These were clearly reflected in resource exploitation provinces, such as Heilongjiang and Shanxi (Figure 5). It was also determined that provincial total Σ_16_PAHs in surface soil and the per capita GDP index levels were negatively correlated (R^2^ = 0.7159) (Figure 8).

These data indicate that total regional levels of PAHs in soils were possibly related to the development and transformation of the economic structure in this country. With per capita GDP values in China approaching those corresponding to high-income countries, the internal driving force of the Chinese economy changed. China is at a point of transition between a stage of economic growth with high consumption and high pollution, to one based on a green economy with high energy and resource efficiency and low environmental loading [106]. With an increasing environmental awareness, some coal and oil mining companies and power plants are using new technologies to mitigate pollutant emissions during the process of energy extraction and use [107]. The energy structure in China transitioned from a stage mainly based on coal and petroleum to one with multiple energy sources, including coal, petroleum, gas, and others. Considering the significant correlation between the provincial total Σ_16_PAHs in surface soils and the total production and consumption of crude oil and coal (*r* = 0.492, *p* < 0.01) (Table 1), six provinces (Inner Mongolia, Shaanxi, Hebei, Liaoning, Jiangsu, Guangdong) were selected and compared. The results demonstrate that the provincial total Σ_16_PAHs in surface soils decreased with decreasing levels of crude oil and coal production and consumption (1997–2016) (Figure 8).

### 4.4. Limitations and Uncertainty Analysis

First, the uneven spatial distribution of the collected sampling sites and differences in sampling methods in the collected references may affect the consistency of the PAH concentration. Statistical references from northeast to southwest regions with a large span from north to south (about 25 of altitude) were involved in the study. Soils in northern China included many more sampling sites, whereas the center and southern regions contained few sampling sites. Thus, places with more soil samples might have a greater contribution to the PAH concentrations at a national scale. Then, a sample number was taken as the weight to calculate the PAH sampling site concentration (sample-number-weighted mean (*u*)) and was used as the spatial interpolation.

**Figure 9:**
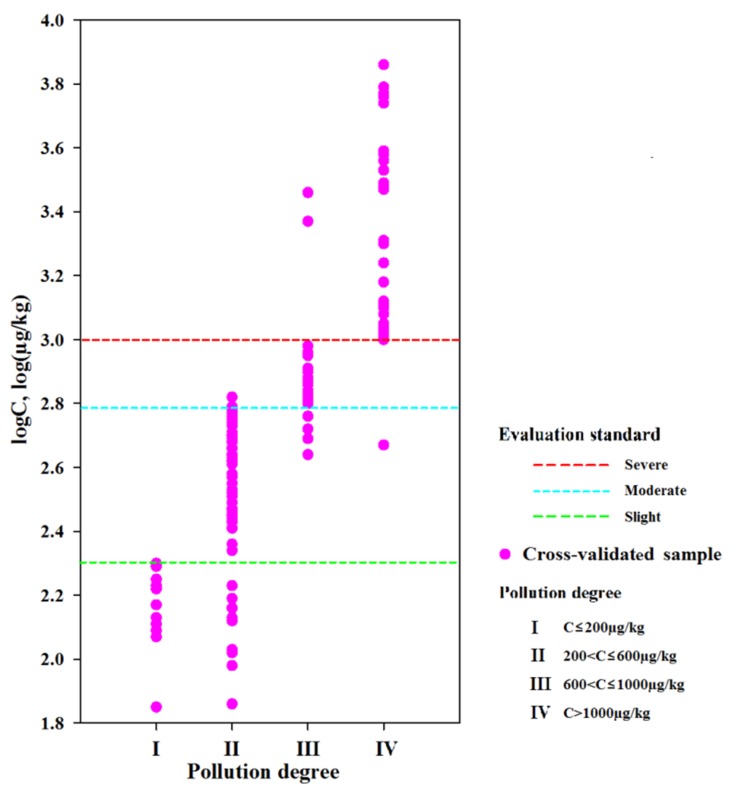
The cross-validation result of spatial distribution using data from 130 new sample sites.

Second, in the present work, contamination data of PAHs in soil samples were obtained using the bibliometrics method [46]. Thus, data might be influenced by the researchers, who were prone to selecting heavily contaminated soil points [13,15,108,109]. The extremely high PAH concentrations were usually caused by mining or industry activities, which are important for small-scale research, because they might reflect the true state of regional soil pollution. However, those heavily contaminated sites might not be representative of the real PAH contamination, and they cannot be used to map spatial variations of PAHs at a national scale, because they might result in an overestimation of the soil PAH levels. In order to avoid such overestimation, and to enhance the spatial interpolation accuracy, the abnormal values were artificially removed in the range of [x/4, 4x]. This study cross-validated the spatial distribution of PAHs in soils by using the selected samples from the new dataset, including 130 sample sites (Figure 9). The results demonstrated that only 20 cross-validated samples’ soil PAH concentrations were not within the corresponding spatial interpolation result of the contamination levels. Furthermore, the soil PAH concentration in the spatial distribution and that from the 31 new reports and 130 sampling sites, about 2080 sample points, showed strong positive and significant correlations (Pearson’s *r* = 0.676, *p* < 0.01) (Table S2). This concordance between the interpolation results and independent results obtained from the data of the 31 new reports is compelling. Given the PAH properties, local scale variation in the soil environment, and the fact that this study’s data and the 31 new reports’ data were independently generated by using completely different methods, these results verify that the interpolation results of the soil PAHs’ spatial distribution are reliable.

Third, the migration, transformation, and accumulation processes of PAHs in the entire environmental system are relatively complicated. China covers a large territory with complex soil types, so the geographical positions, terrains, climatic, light, and humidity conditions in different regions were significantly different [110,111,112]. Besides, the energy structure was also very different. North China uses large amounts of coal combustion for heating during the winter [113,114]. Therefore, the PAH concentration, type, and individual PAH representatives in surface soil displayed large variation in different regions. All of these factors were found to influence the processes of PAH generation, diffusion, main sources, and distribution, as well as their accumulation and adsorption in the soil media [114,115,116]. Furthermore, each source apportionment and receptor-oriented approach has its own advantages and disadvantages. Even with the application of multiple techniques (diagnostic ratios, PCA-MLR model, and Pearson correlation coefficient) to minimize the bias of each single method, it is still necessary to construct a comprehensive monitoring system on PAHs in soils to master the real-time status of soil PAH pollution in future research.

## 5. Conclusions

In this work, the spatial occurrence level and distribution of soil PAHs in China were evaluated. In addition, the relationship between the PAH levels in soils and anthropogenic activities was investigated. Our results showed that the concentration of PAHs in surface soils in China presented a descending gradient in the order of IMIA > urban area > suburban area > rural area. The PAH concentration in northern China was far higher than in the south. The source analysis of soil PAHs demonstrated that the main causes of PAHs are coal combustion and vehicular exhaust emissions. Besides, the per square kilometer GDP of industrial land, the per capita GDP, and the exploitation and use of coal and petroleum are closely related to the provincial total Σ_16_PAHs in surface soil. Until now, systematic and large-scale studies on PAH contamination in surface soil have been scarce. In addition, the current soil environmental quality standards do not consider PAHs as a parameter. Thus, comprehensive monitoring of PAHs in soils should be periodically conducted in the future to confirm the temporal and spatial characteristics and mechanism of PAH pollution. Also, it is necessary to set up environmental standards for PAHs in soils and provide scientific evidence that can help in contamination management and the formulation of proper policies.

## Figures and Tables

**Table 1:** Pearson correlation coefficients for PAHs and energy indicators.

Item	LMW	HMW	Provincial Total Σ_16_PAHs	Petroleum Reserves	Natural Gas Reserves	Coal Reserves	ΣP-Coal	ΣP&C-Crude Oil and Coal
LMW	1							
HMW	−0.023 (*n* = 31)	1						
Provincial total Σ_16_PAHs	0.410 * (*n* = 31)	0.521 ** (*n* = 31)	1					
Petroleum reserves	0.695 ** (*n* = 22)	0.155 (*n* = 22)	0.476 * (*n* = 22)	1				
Natural gas reserves	0.101 (*n* = 24)	0.571 ** (*n* = 24)	0.643 ** (*n* = 24)	0.327 (*n* = 24)	1			
Coal reserves	−0.009 (*n* = 30)	0.655 ** (*n* = 30)	0.491 ** (*n* = 30)	0.234 (*n* = 22)	0.226 (*n* = 24)	1		
ΣP-coal	−0.048 (*n* = 30)	0.677 ** (*n* = 30)	0.540 ** (*n* = 30)	0.190 (*n* = 21)	0.281 (*n* = 23)	0.956 ** (*n* = 29)	1	
ΣP&C-crude oil and coal	−0.006 (*n* = 30)	0.565 ** (*n* = 30)	0.492 ** (*n* = 30)	0.276 (*n* = 21)	0.187 (*n* = 23)	0.873 ** (*n* = 29)	0.940 ** (*n* = 30)	1

Note: ΣP-coal means total production of coal from 1997 to 2016 in China; ΣP&C-crude oil and coal means total production and consumption of crude oil and coal from 1997 to 2016 in China; * means significant correlation at the 0.05 level; ** means significant correlation at the 0.01 level. Detailed data information of this table’s data can be found in the Supplementary Material.

**Table 2:** Comparison of PAH concentration (µg/kg) observed in this study with those found in other regions of the world in different soil types.

	Urban	Suburban	Rural	IMIA
China	1479.97 (0.02–39,891)	523.34 (3.51–6248)	293.95 (0.38–5910)	8800.77 (5.1–97,680)
Delaware, USA ^a^	2732 (362–16462)	172 (70–448)	170 (78.7–497)	-
Delhi and Bajalkota, India ^b^	5524.25 (1550.9–11,460)	-	1458 (827–2095)	3920 (1190–12,700)
Izmir and Bursa, Turkey ^c^	2243.5	55.9	152	4628
London and England, UK ^d^	68,000	41,000	590	-
Ulsan, Korea and South Korea ^e^	390 (65–1200)	236 (23.3–2834)	220 (92–450)	2515

Note: Detailed information about references a, b, c, d, and e of this table can be found in the Supplementary Material.

## Supplementary Materials

The following are available online at https://www.mdpi.com/1660-4601/16/24/4928/s1, Table S1: PAHs concentrations (µg/kg) of soil sampling points in China; Table S2: The data from 130 new sample sites for cross-validation of spatial distribution.
