# Supplementary material for: Assessing the Spatial Distribution of Soil PAHs and their Relationship with Anthropogenic Activities at a National Scale
Source: Int J Environ Res Public Health. 2019 Dec 5;16(24):4928. doi: 10.3390/ijerph16244928 (PMC6950367; doi:10.3390/ijerph16244928)
Supplement: Supplementary file 1 [file ijerph-16-04928-s001.zip › ijerph-635121-Table S2.docx]

**Table S2.** The data from 130 new sample sites for cross-validation of spatial distribution.

| **No.** | **Longitude** | **Latitude** | **Province/Fields** | **ΣPAHs**  **(Raw Data)** | **Reference** | **Number of Samples** | **Interpolated Value** |
| --- | --- | --- | --- | --- | --- | --- | --- |
| 1 | 110.4824 | 25.5749 | Guangxi/Jinlin City, Xing'an County, Rongjiang Town | 72.81 | Xie et al.,2019 | 19 | 569.318542 |
| 2 | 111.5022 | 24.3767 | Guangxi/Hezhou City (aquatic Vegetable soil) | 1255.60 | Zhao et al.,2019 | 20 | 1123.236816 |
| 3 | 111.5597 | 24.3656 | Guangxi/Hezhou City (Lusheng Vegetable Soil) | 1078.53 |  | 20 | 1123.236816 |
| 4 | 110.8217 | 24.6642 | Guangxi/Guilin City (Aquatic Vegetable soil) | 1055.48 |  | 20 | 1123.236816 |
| 5 | 110.8517 | 24.5956 | Guangxi/Guilin City (Lusheng Vegetable Soil | 918.57 |  | 20 | 912.257446 |
| 6 | 110.5050 | 24.5378 | Guangxi/Zhangpu City (Aquatic Vegetable soil) | 1306.95 |  | 20 | 1220.681396 |
| 7 | 110.4350 | 24.6031 | Guangxi/Zhangpu City (Lusheng Vegetable Soil) | 951.40 |  | 20 | 906.419189 |
| 8 | 108.3325 | 22.9997 | Guangxi/Nanning City (Aquatic Vegetable soil) | 1282.91 |  | 20 | 1803.893066 |
| 9 | 108.4975 | 22.7097 | Guangxi/Nanning City (Lusheng Vegetable Soil) | 1044.40 |  | 20 | 1088.442993 |
| 10 | 104.2934 | 30.8797 | Sichuan/Chengdu Qingbaijiang Industrial Development Zone | 1050.30 | Guo et al.,2019 | 57 | 1404.23645 |
| 11 | 114.0669 | 22.5541 | Guangdong/Shenzhen Opencast Planting Area | 171.38 | Liu et al.,2019 | 36 | 111.417709 |
| 12 | 107.1116 | 29.8816 | Chongqing/Open planting area | 147.97 |  | 36 | 156.47435 |
| 13 | 121.4921 | 29.3983 | Zhejiang/Ningbo Opencast Planting Area | 135.60 |  | 36 | 173.108688 |
| 14 | 120.4593 | 36.9969 | Shandong/Qingdao, Forest soil along the Dagu River | 330.00 | Cheng et al.,2018 | 1 | 380.323822 |
| 15 | 120.0137 | 36.4812 | Shandong/Qingdao, Jiaozhou area, Vegetable base soil | 648.60 |  | 1 | 646.339294 |
| 16 | 119.4296 | 36.0205 | Shandong/Weifang Zhucheng, Farmland soil around Thermal Power Plant | 2354.80 |  | 1 | 646.81958 |
| 17 | 113.2456 | 23.0910 | Guangdong/Pearl River Delta (Constructed Wetland) | 640.06 | Han et al.,2019 | 6 | 793.592468 |
| 18 | 113.4204 | 22.9164 | Guangdong/Pearl River Delta (Urban River Wetlands) | 626.15 |  | 6 | 628.25708 |
| 19 | 113.4457 | 22.8546 | Guangdong/Pearl River Delta (Rural River Wetland) | 740.12 |  | 6 | 615.632568 |
| 20 | 104.0611 | 30.7085 | Sichuan/Chengdu Jinniu District (Lubricating Oil Factory 1) | 1005.20 | Ran et al.,2019 | 4 | 1782.006714 |
| 21 | 104.1106 | 30.7761 | Sichuan/Chengdu Jinniu District (Lubricating Oil Factory 2) | 369.80 |  | 4 | 497.86087 |
| 22 | 104.2520 | 30.8902 | Sichuan/Chengdu Qingbaijiang District (Asphalt Plant 1) | 476.00 |  | 4 | 497.86087 |
| 23 | 103.8157 | 30.4191 | Sichuan/Chengdu Xinjin County (Asphalt Plant 2) | 548.50 |  | 4 | 551.09552 |
| 24 | 121.5878 | 31.0134 | Shanghai/Farmland soil on both sides of traffic trunk line | 353.77 | Zhang,2019 | 112 | 337.680481 |
| 25 | 125.3699 | 43.7187 | Jilin/Changchun City, Farmland soil along the roads | 2954.93 | Chen,2019 | 35 | 1712.44165 |
| 26 | 125.5363 | 44.3386 | Jilin/Dehui City, Farmland soil along major highways | 1529.48 |  | 108 | 1405.609985 |
| 27 | 121.4667 | 31.4152 | Shanghai/Baoshan Baoshan Iron and Steel Plant (BS) | 535.30 | Jia,2019 | 201 | 554.401062 |
| 28 | 121.7184 | 31.0892 | Shanghai/Pudong New Area (PD) | 421.65 |  |  | 436.049103 |
| 29 | 121.5155 | 30.9177 | Near Shanghai/Fengxian District Hujin Expressway (FX) | 294.78 |  |  | 554.401062 |
| 30 | 121.1115 | 31.1535 | Shanghai/Qingpu District (control) (QP) | 258.05 |  |  | 538.418701 |
| 31 | 121.4663 | 31.0819 | Shanghai/Minhang District Wujing Chemical Factory (MH1) | 372.74 |  |  | 337.680481 |
| 32 | 121.4663 | 31.0819 | Shanghai/Minhang District Wusong Chemical Factory (MH2) | 308.66 |  |  | 337.680481 |
| 33 | 121.4625 | 31.0805 | Shanghai/Minhang District Wujing Chemical Factory (MH3) | 270.86 |  |  | 337.680481 |
| 34 | 121.4580 | 31.0780 | Shanghai/Minhang District Wujing Chemical Factory (MH4) | 454.42 |  |  | 337.680481 |
| 35 | 115.6169 | 39.6922 | Beijing/Suburb (farmland soil) | 460.75 | Zhou et al.,2019 | 168 | 533.0401 |
| 36 | 116.4440 | 40.1488 | Beijing/Changping | 517.24 |  | 10 | 526.447083 |
| 37 | 116.2558 | 39.5852 | Beijing/Daxing | 565.52 |  | 25 | 229.148865 |
| 38 | 116.0029 | 39.8390 | Beijing/Fangshan | 406.90 |  | 18 | 343.714081 |
| 39 | 116.3934 | 39.9242 | Beijing/Chaoyang | 468.97 |  | 5 | 1192.119751 |
| 40 | 116.3073 | 40.0442 | Beijing/Haidian | 1068.97 |  | 5 | 1192.119751 |
| 41 | 116.6161 | 40.3082 | Beijing/Huairou | 510.35 |  | 5 | 344.120575 |
| 42 | 116.8478 | 40.4820 | Beijing/Miyun | 289.66 |  | 12 | 396.700195 |
| 43 | 116.6446 | 40.2647 | Beijing/Shunyi | 462.07 |  | 20 | 344.120575 |
| 44 | 116.7052 | 39.7321 | Beijing/Tongzhou | 441.38 |  | 20 | 623.084839 |
| 45 | 116.1562 | 40.6093 | Beijing/Yanqing | 275.86 |  | 5 | 326.715302 |
| 46 | 106.6544 | 29.6629 | Chongqing/Majiagou Coal Mine | 170.30 | Sun et al.,2019 | 18 | 244.847992 |
| 47 | 119.3392 | 35.1765 | Shandong/Rizhao City, Lanshan chemical plant, Laoshan District | 3089.80 | Guo et al.,2018 | 10 | 1089.989624 |
| 48 | 118.3097 | 34.9600 | Shandong/Linyi City, Huayu electrolytic aluminum plant | 2911.40 |  | 10 | 900.561279 |
| 49 | 98.7224 | 34.2317 | Qinghai/the Qingzang Highway | 267.97 | Zhou et al.,2018 | 55 | 268.537811 |
| 50 | 103.5805 | 36.1904 | Gansu/Lanzhou | 5734.00 | Ding et al.,2018 | 9 | 2698.046631 |
| 51 | 121.4778 | 31.4087 | Shanghai/Steel Industrial Zone (close-up sample area) | 1126.50 | Qi et al.,2018 | 3 | 2592.41748 |
| 52 | 121.4519 | 31.4064 | Shanghai/Steel Industrial Zone (middle distance sample area) | 659.90 |  | 6 | 877.891785 |
| 53 | 121.3475 | 31.3786 | Shanghai/Steel Industrial Zone (distance sample area) | 165.80 |  | 5 | 183.898941 |
| 54 | 123.4078 | 41.8357 | Liaoning/Shenyang Baibird Park | 885.42 | Liu et al.,2018 | >3 | 681.291199 |
| 55 | 123.4354 | 41.8576 | Liaoning/Shenyang Beiling Park | 2031.25 |  | >3 | 1487.109497 |
| 56 | 123.4811 | 41.7965 | Liaoning/Shenyang Wanquan Park | 3906.25 |  | >3 | 1150.973145 |
| 57 | 123.4203 | 41.7768 | Liaoning/Shenyang Nanhu Park | 1718.75 |  | >3 | 1602.915283 |
| 58 | 123.4128 | 41.7913 | Liaoning/Shenyang Zhongshan Park | 5833.33 |  | >3 | 1602.915283 |
| 59 | 123.3708 | 41.7942 | Liaoning/Shenyang Xinghua Park | 312.50 |  | >3 | 342.961731 |
| 60 | 123.4464 | 41.7872 | Liaoning/Shenyang Youth Park | 3593.75 |  | >3 | 1602.915283 |
| 61 | 121.5927 | 31.3599 | Shanghai/Power Plant (P1) | 2005.45 | Wu,2018 | 1 | 2592.41748 |
| 62 | 121.6044 | 31.3499 | Shanghai/Power Plant (P2) | 5548.01 |  | 1 | 2592.41748 |
| 63 | 121.4007 | 30.7612 | Shanghai/Power Plant (P3) | 572.94 |  | 1 | 943.808594 |
| 64 | 121.5565 | 31.2736 | Shanghai/Power Plant (P4) | 6223.52 |  | 1 | 2592.41748 |
| 65 | 121.4038 | 31.4743 | Shanghai/Power Plant (P5) | 755.38 |  | 1 | 705.254211 |
| 66 | 121.4089 | 31.4610 | Shanghai/Power Plant (P6) | 787.83 |  | 1 | 705.254211 |
| 67 | 121.5294 | 31.3466 | Shanghai/Power Plant (P7) | 3389.74 |  | 1 | 2592.41748 |
| 68 | 121.4591 | 31.4080 | Shanghai/Power Plant (P8) | 280.28 |  | 1 | 436.049103 |
| 69 | 121.4642 | 31.0614 | Shanghai/Power Plant (P9) | 306.34 |  | 1 | 337.680481 |
| 70 | 121.3276 | 30.7197 | Shanghai/Industrial Park (I1) | 7302.68 |  | 1 | 1136.097412 |
| 71 | 121.4654 | 30.8209 | Shanghai/Industrial Park (I2) | 131.66 |  | 1 | 554.401062 |
| 72 | 121.4983 | 30.8988 | Shanghai/Industrial Park (I3) | 336.71 |  | 1 | 554.401062 |
| 73 | 121.5918 | 31.2123 | Shanghai/Industrial Park (I4) | 493.71 |  | 1 | 337.680481 |
| 74 | 121.2661 | 31.0149 | Shanghai/Industrial Park (I5) | 697.56 |  | 1 | 877.891785 |
| 75 | 121.1369 | 31.1791 | Shanghai/Industrial Park (I6) | 427.85 |  | 1 | 407.144775 |
| 76 | 121.2344 | 31.3615 | Shanghai/Industrial Park (I7) | 228.53 |  | 1 | 538.418701 |
| 77 | 121.3845 | 31.0050 | Shanghai/Industrial Park (I8) | 524.21 |  | 1 | 943.808594 |
| 78 | 121.4174 | 31.4113 | Shanghai/Environmental Protection Company (E1) | 1090.01 |  | 1 | 2592.41748 |
| 79 | 121.1806 | 31.4527 | Shanghai/Environmental Protection Company (E2) | 153.78 |  | 1 | 347.151276 |
| 80 | 121.1739 | 31.4461 | Shanghai/Environmental Protection Company (E3) | 170.13 |  | 1 | 347.151276 |
| 81 | 121.8610 | 31.0431 | Shanghai/Environmental Protection Company (E4) | 231.04 |  | 1 | 436.049103 |
| 82 | 121.7334 | 31.2106 | Shanghai/Environmental Protection Company (E5) | 439.12 |  | 1 | 436.049103 |
| 83 | 121.4254 | 30.8242 | Shanghai/Environmental Protection Company (E6) | 176.39 |  | 1 | 183.898941 |
| 84 | 121.2655 | 30.7977 | Shanghai/Environmental company (E7) | 419.75 |  | 1 | 554.401062 |
| 85 | 121.2895 | 30.9804 | Shanghai/Green Belt | 1048.72(Σ_15_PAHs) | Li,2018 | 12 | 1095.849365 |
| 86 | 120.9045 | 31.0721 | Shanghai/Farmland | 198.77(Σ_15_PAHs) |  | 12 | 183.898941 |
| 87 | 111.4137 | 36.0684 | Shanxi/Linyi City | 723.20 | Tao et al.,2016 | 128 | 661.682312 |
| 88 | 88.9575 | 44.9100 | Xinjiang/Jimsar County Colorful Bay (sample1) | 493.00 | Zhang et al.,2017 | 3 | 753.489685 |
| 89 | 89.1544 | 44.9395 | Xinjiang/Jimsar County Colorful Bay (sample2) | 743.00 |  | 3 | 697.606567 |
| 90 | 89.1892 | 44.9531 | Xinjiang/Jimsar County Colorful Bay (sample3) | 433.00 |  | 3 | 598.851563 |
| 91 | 89.2085 | 44.9284 | Xinjiang/Jimsar County Colorful Bay (sample4) | 106.00 |  | 3 | 598.851563 |
| 92 | 89.1371 | 44.9095 | Xinjiang/Jimsar County Colorful Bay (sample5) | 731.00 |  | 3 | 697.606567 |
| 93 | 89.1718 | 44.8670 | Xinjiang/Jimsar County Colorful Bay (sample6) | 134.00 |  | 3 | 553.961304 |
| 94 | 111.3598 | 41.1758 | Inner Mongolia/Hohhot | 338.00 | Zhang and Zhang,2017 | 60 | 398.459839 |
| 95 | 114.3091 | 23.0151 | Guangdong/Huizhou City | 123.09 | Wang et al.,2017a | 42 | 130.343628 |
| 96 | 117.4179 | 38.8384 | Tianjin | 3370.00 | Li et al.,2010 | 188 | 4743.452148 |
| 97 | 117.4042 | 38.6014 | Tianjin/Dagang Oilfield Arable Desert Soil Area(S0) | 372.80 | Jiao et al.,2015 | 1 | 383.479523 |
| 98 | 117.1487 | 38.6038 | Tianjin/Dagang Oilfield Arable Desert Soil Area (S1) | 103.60 |  | 1 | 222.100998 |
| 99 | 117.4048 | 38.6567 | Tianjin/Dagang Oilfield Arable Desert Soil Area (S2) | 505.30 |  | 1 | 572.431885 |
| 100 | 117.4056 | 38.6720 | Tianjin/Dagang Oilfield Arable Desert Soil Area (S3) | 143.20 |  | 1 | 572.431885 |
| 101 | 117.4130 | 38.6592 | Tianjin/Dagang Oilfield Arable Desert Soil Area (S4) | 650.30 |  | 1 | 731.727661 |
| 102 | 117.4146 | 38.6686 | Tianjin/Dagang Oilfield Arable Desert Soil Area (S5) | 556.80 |  | 1 | 572.431885 |
| 103 | 117.1970 | 38.6147 | Tianjin/Dagang Oilfield Arable Desert Soil Area (S17) | 117.50 |  | 1 | 168.737167 |
| 104 | 117.3232 | 38.5948 | Tianjin/Dagang Oilfield Arable Desert Soil Area (S20) | 196.60 |  | 1 | 168.737167 |
| 105 | 117.4312 | 38.6200 | Tianjin/Dagang Oilfield Arable Desert Soil Area (S21) | 492.90 |  | 1 | 572.431885 |
| 106 | 117.3972 | 38.7111 | Tianjin/Dagang Oilfield Arable Desert Soil Area (S22) | 289.20 |  | 1 | 572.431885 |
| 107 | 117.3288 | 38.6135 | Tianjin/Dagang Oilfield Area oil well areas(S9) | 593.80 |  | 1 | 572.431885 |
| 108 | 117.3820 | 38.6355 | Tianjin/Dagang Oilfield Arable Desert Soil Area (S10) | 735.60 |  | 1 | 731.727661 |
| 109 | 117.4810 | 38.6607 | Tianjin/Dagang Oilfield Area oil well areas(S12) | 817.40 |  | 1 | 731.727661 |
| 110 | 117.5029 | 38.7134 | Tianjin/Dagang Oilfield Arable Desert Soil Area (S13) | 601.60 |  | 1 | 572.431885 |
| 111 | 117.2151 | 38.5883 | Tianjin/Dagang Oilfield Area oil well areas(S16) | 129.30 |  | 1 | 130.343628 |
| 112 | 117.5592 | 38.7455 | Tianjin/Dagang Oilfield Arable Desert Soil Area (S23) | 217.00 |  | 1 | 222.100998 |
| 113 | 117.5446 | 38.7209 | Tianjin/Dagang Oilfield Area oil well areas(S24) | 340.60 |  | 1 | 383.479523 |
| 114 | 117.5610 | 38.7056 | Tianjin/Dagang Oilfield Arable Desert Soil Area (S25) | 1214.00 |  | 1 | 1494.038574 |
| 115 | 117.5071 | 38.6836 | Tianjin/Dagang Oilfield Area oil well areas(S26) | 1029.00 |  | 1 | 1494.038574 |
| 116 | 117.3597 | 38.6721 | Tianjin/Dagang Oilfield Resident (S6) | 618.10 |  | 1 | 572.431885 |
| 117 | 117.3377 | 38.6551 | Tianjin/Dagang Oilfield Resident (S7) | 572.50 |  | 1 | 572.431885 |
| 118 | 117.2772 | 38.6281 | Tianjin/Dagang Oilfield Resident (S8) | 1021.00 |  | 1 | 1494.038574 |
| 119 | 117.4335 | 38.6524 | Tianjin/Dagang Oilfield Resident (S11) | 3783.00 |  | 1 | 4743.452148 |
| 120 | 117.4349 | 38.7195 | Tianjin/Dagang Oilfield Resident (S14) | 661.10 |  | 1 | 572.431885 |
| 121 | 117.2308 | 38.5611 | Tianjin/Dagang Oilfield Resident (S15) | 2022.00 |  | 1 | 4743.452148 |
| 122 | 117.2845 | 38.6553 | Tianjin/Dagang Oilfield Resident (S18) | 335.10 |  | 1 | 572.431885 |
| 123 | 117.3059 | 38.6238 | Tianjin/Dagang Oilfield Resident (S19) | 5872.00 |  | 1 | 4743.452148 |
| 124 | 113.0167 | 23.4193 | Guangdong/Longtang(rhizosphere soils) | 415.00 | Wang et al.,2017b | 33 | 473.0448 |
| 125 | 113.0000 | 23.5667 | Guangdong/Longtang(non-rhizosphere soils) | 322.00 |  | 33 | 469.082977 |
| 126 | 116.8000 | 35.3800 | Shangdong/Zoucheng, the Rong Xin coking plant | 3016.00 | Wu et al.,2018 | 17 | 1067.045044 |
| 127 | 123.5912 | 45.9436 | Jilin/Zhenlai County, The Momoge Nature Reserve | 96.00 | Xu et al.,2017 | 39 | 472.885956 |
| 128 | 117.8299 | 37.8221 | Shandong/the border regions between the oil fields and suburbs in soils of the YRD | 382.50 | Fu et al.,2018 | 138 | 570.058044 |
| 129 | 118.5096 | 37.9373 | Shandong/the Northern Yellow River Delta | 71.10 | Yuan et al.,2013 | 20 | 33.699097 |
| 130 | 121.6343 | 41.0059 | Liaoning/Panjin City, Liaohe estuarine wetland surface soils | 675.40 | Wang et al.,2019 | 31 | 724.418579 |

References:

1. Chen Y. Risk assessment of polycyclic aromatic hydrocarbons in soil and maize along roadside. Northeast Normal University (Doctoral dissertation). 2019.
2. Cheng Q, Guo J, Chai C, et al. Accumulation characteristics and health risk assessment of polycyclic aromatic hydrocarbons in spinach grown in diesel contaminated soil. Journal of Ecology and Rural Environment 2018; 34, 1145-1152.
3. Ding H, Tao X, Lv K, et al. Distribution characteristics and risk analysis of PAHs and PCBs in soils of Lanzhou. The Administration and Technique of Environmental Monitoring 2018; 30, 25-29.
4. Fu X, Li T, Ji L, et al. Occurrence, sources and health risk of polycyclic aromatic hydrocarbons in soils around oil wells in the border regions between oil fields and suburbs. Ecotoxicology and Environmental Safety 2018; 157, 276-284.
5. Guo J, Ge W, Chao C, et al. Concentrations, sources, and health risk of polycyclic aromatic hydrocarbons in soils around chemical plants. Environmental Chemistry 2018; 37, 296-309.
6. Guo X. Identification of polycyclic aromatic hydrocarbon pollution sources and ecological risk assessment of soils in typical industrial parks in Chengdu. Environmental Engineering 2019; 37, 950-956.
7. Han L, Gao Z, Bai J, et al. PAHs in surface wetland soils of the Pearl River Delta affected by urbanization: levels, sources, and toxic risks. Journal of Agro-Environment Science 2019; 38, 609-617.
8. Jia J. Accumulation characteristics and exposure risk of PAHs in air-leaf vegetable-soil system: a case study in Shanghai suburbs. East China Normal University (Doctoral dissertation). 2019.
9. Jiao H, Rui X, Wu S, et al. Polycyclic aromatic hydrocarbons in the Dagang Oilfield (China): distribution, sources, and risk assessment. International Journal of Environmental Research and Public Health 2015; 12, 5775-5791.
10. Li D. Distribution and ecological risk assessment of polycyclic aromatic hydrocarbons (PAHs) in agricultural soil of roadside. East China University of Science and Technology (Doctoral dissertation). 2018.
11. Li X, Tao S, Liu W, et al. Dry deposition of polycyclic aromatic hydrocarbons and its influence on surface soil contamination in Tianjin, China. Journal of Environmental Monitoring 2010; 12, 952-957.
12. Liu W, Liu W, Zhang W, et al. Study on the soil PAHs pollution characteristics of main parks in Shenyang. Journal of Meteorology and Environment 2018; 34, 69-74.
13. Liu Y. Investigation of residual polycyclic aromatic hydrocarbons in soil-vegetable and fruit system in vegetable cultivation area of Shenzhen Chongqing and Ningbo. Shandong Chemical Industry 2019; 48, 240-243.
14. Qi X, Huang S, Sha C, et al. Pollution characteristics and source apportionment of polycyclic aromatic hydrocarbons in surface soil of the steel industrial downwind area. Research of Environmental Sciences 2018; 31, 927-934.
15. Ran Z, Chen J, Wang Y, et al. Characteristics and influencing factors of polycyclic aromatic hydrocarbons in surface soils from typical industrial areas of Chengdu. Environmental Science 2019; 40, 4594-4603.
16. Sun X, Wang F, Guo T, et al. Occurrence and risk assessment of polycyclic aromatic hydrocarbons in topsoil of an abandoned coal mine area in Chongqing. Earth and Environment 2019; 47, 502-509.
17. Tao S, Ma J, Zhou Y, et al. Polycyclic aromatic hydrocarbons pollution an d risk assessment in soil of typical coal-fired pollution region in Shanxi province. Ecology and Environmental Sciences 2016; 25, 2005-2013.
18. Wang K, Yang G, Wang Y. A study on soil PAHs spatial distribution characters of Huizhou city based on GIS. Ecology and Environmental Sciences 2017a; 26, 700-707.
19. Wang N, Lang Y, Cheng F, et al. Concentrations, sources and risk assessment of polycyclic aromatic hydrocarbons (PAHs) in soils of Liaohe Estuarine Wetland. Bulletin of Environmental Contamination and Toxicology 2011; 87, 463-468.
20. Wang Y, He J, Wang S, et al. Characterisation and risk assessment of polycyclic aromatic hydrocarbons (PAHs) in soils and plants around e-waste dismantling sites in southern China. Environmental Science and Pollution Research 2017b; 24, 22173–22182.
21. Wu J, Li K, Ma D, et al. Contamination, source identification, and risk assessment of polycyclic aromatic hydrocarbons in agricultural soils around a typical coking plant in Shandong, China. Human and Ecological Risk Assessment 2018; 24, 225-241.
22. Wu S. Spatial differentiation and influencing factors of PAHs in urban soil columns of Shanghai. East China Normal University (Doctoral dissertation). 2018.
23. Xie Y, Tang X, Chen Y. Content and sources of PAHs in topsoils of orchards in Guilin. Southwest China Journal of Agricultural Sciences 2019; 32, 1825-1832.
24. Xu J, Wang H, Sheng L, et al. Distribution characteristics and risk assessment of polycyclic aromatic hydrocarbons in the Momoge Wetland, China. International Journal of Environmental Research and Public Health 2017; 14, 85-99.
25. Yuan H, Li T, Ding X, et al. Distribution, sources Analysis and eco-toxicological risk assessment of polycyclic aromatic hydrocarbons (PAHs) in surface soils in the northern Yellow River Delta, China. Advanced Materials Research 2013; 726-731,750-756.
26. Zhang L, Chen Y, Kong L, et al. Analysis of pollution characteristics of PAHs in Xinjiang Zhundong coal mining area. Environmental Chemistry 2017; 36, 677-684.
27. Zhang X, Zhang F. Pollution characteristics and ecological risk assessment of polycyclic aromatic hydrocarbons in agricultural soils of Huhhot, China. Agricultural Science and Technology 2017; 18, 747-752.
28. Zhang X. PAHs distribution characteristics, source analysis and risk assessment in farmland soil along Shanghai traffic line. East China Normal University (Doctoral dissertation). 2019.
29. Zhao T, Long M, Qiao S, et al. Pollution characteristics of polycyclic aromatic hydrocarbons in aquatic vegetable soils of Guangxi province. Earth and Environment 2019; 47, 728-737.
30. Zhou J, Zhang J, Liu X, et al. Pollution characteristics and risk assessment of PAHs in agricultural soil in suburb of Beijing. Journal of Agricultural Resources and Environment 2019; 36, 534-540.
31. Zhou W, Li J, Hu J, et al. Distribution, sources, and ecological risk assessment of polycyclic aromatic hydrocarbons (PAHs) in soils of the central and eastern areas of the Qinghai-Tibetan Plateau. Environmental Science 2018; 39, 1413-1420.
