# Supplementary material for: Assessing the Spatial Distribution of Soil PAHs and their Relationship with Anthropogenic Activities at a National Scale
Source: Int J Environ Res Public Health. 2019 Dec 5;16(24):4928. doi: 10.3390/ijerph16244928 (PMC6950367; doi:10.3390/ijerph16244928)
Supplement: Supplementary file 1 [file ijerph-16-04928-s001.zip › Table S/Tables.docx]

**Tables:**

**Table S1.** PAHs concentrations (µg/kg) of soil sampling points in China

**Table 1.** PCA-MLR model of PAHs data for surface soil samples

**Table 2.** Pearson correlation coefficients for PAHs and energy indicators

**Table 3.** Comparison of PAHs concentration (µg/kg) observed in this study with those found in other region of the world in different soil types

#### **Table S1.** PAHs concentrations (µg/kg) of soil sampling points in China

**The data of PAHs concentration can be found in the Excel table “Table S1”.**

**References:**

An Y, Huang Y, Sun Z, et al. Source apportionment and risk assessment of PAHs in soil from a renewal area in the Tongzhou District of Beijing. Hydrogeology and Engineering Geology 2017; 44: 112-120.

Bao H, Hou S, Niu H, Tian K, Liu X, Wu F. Status, sources, and risk assessment of polycyclic aromatic hydrocarbons in urban soils of Xi’an, China. Environmental Science and Pollution Research 2018; 25: 18947-18959.

Bao X. Study on environmental quality assessment and ecological geochemistry regionalization of topsoil in Jilin-Changchun-Siping urban economic zone. Jilin University(Doctoral dissertation). 2011.

Cai C, Zhang Y, Reid B, et al. Carcinogenic potential of soils contaminated with polycyclic aromatic hydrocarbons (PAHs) in Xiamen metropolis, China. Journal of Environmental Monitoring 2012; 14: 3111-3117.

Cai Q, Mo C, Li Y, et al. Occurrence and assessment of polycyclic aromatic hydrocarbons in soils from vegetable fields of the Pearl River Delta, South China. Chemosphere 2007; 68: 159-168.

Cai X, Xue D, Li D, et al. Distribution and sources of polycyclic aromatic hydrocarbons in the sediments of Ramsar swamp in inner deep bay of Hong Kong. Environmental Chemistry 2013; 32: 451-458.

Cao S, Ni H, Qin P, et al. Occurrence and human non-dietary exposure of polycyclic aromatic hydrocarbons in soils from Shenzhen, China. Journal of Environmental Monitoring 2010; 12: 1445-1450.

Cao X, Liu M, Song Y, Ackland M. Composition, sources, and potential toxicology of polycyclic aromatic hydrocarbons (PAHs) in agricultural soils in Liaoning, People’s Republic of China. Environmental Monitoring and Assessment 2013; 185: 2231-2241.

Cha T. Spatial distribution and risk assessment of polycyclic aromatic hydrocarbons in paddy soil of Wenling. Dissertation of Zhejiang University(Doctoral dissertation). 2015.

Chai C, Cheng Q, Wu J, et al. Contamination, source identification, and risk assessment of polycyclic aromatic hydrocarbons in the soils of vegetable greenhouses in Shandong, China. Ecotoxicology and Environmental Safety 2017; 142: 181-188.

Chen B, Xuan X, Zhu L, et al. Distributions of polycyclic aromatic hydrocarbons in surface waters, sediments and soils of Hangzhou City, China. Water Research 2004a; 38: 3558-3568.

Chen C, Zhang M, Yang J, et al. Pollution status and sources of polycyclic aromatic hydrocarbons in soil of Qiannan state. Ecology and Environmental Sciences 2009a; 18: 929-933.

Chen F, Wang C, Zhang L, et al. Sources appoinmental and risk assessment of polycyclic aromatic hydrocarbon in agricultural soil form zinc smelting area. Acta Sciencetiae Circumstantiae 2017; 37: 1515-1523.

Chen G, Jie Q, Shi D, et al. Diversity of soil nematodes in areas polluted with heavy metals and polycyclic aromatic hydrocarbons (PAHs) in Lanzhou, China. Environmental Management 2009b; 44: 163-172.

Chen L, Ran Y, Mai B, et al. Pollution situation of polynucear aromatic hydrocarbons in Vegetable soils around Guangzhou. Environmental Chemistry 2004b; 23: 341-344.

Chen L, Yong R, Xing B, et al. Contents and sources of polycyclic aromatic hydrocarbons and organochlorine pesticides in vegetable soils of Guangzhou, China. Chemosphere 2005; 60: 879-890.

Chen M, Chen L, Huang P. Concentration and ecological risks of polycyclic aromatic hydrocarbons in the surface soils of Urumqi area, China. Environmental Monitoring in China 2015; 31: 84-91.

Chen Q, Ma J, Guo B. The distribution characterization/sources and risk assessment of PAHs in different agriculture typical areas of Shandong province. Ecology and Environmental Sciences 2016a; 25: 1006-1013.

Chen Y, Zhang F, Zhang J, Zhou M, Li F, Liu X. Accumulation characteristics and potential risk of PAHs in vegetable system grow in home garden under straw burning condition in Jilin, Northeast China. Ecotoxicology and Environmental Safety 2018a; 162: 647-654.

Chen Y, Zhang J, Ma Q, Sun C, Ha S, Zhang F. Human health risk assessment and source diagnosis of polycyclic aromatic hydrocarbons (PAHs) in the corn and agricultural soils along main roadside in Changchun, China. Human and Ecological Risk Assessment 2016b; 22: 706-720

Chen Y, Zhang J, Zhang F, et al. Contamination and health risk assessment of PAHs in farmland soils of the Yinma River Basin, China. Ecotoxicology and Environmental Safety 2018b; 156: 383-390.

Chen Y, Zhang J, Zhang F, Li F, Zhou M. Polycyclic aromatic hydrocarbons in farmland soils around main reservoirs of Jilin Province, China: occurrence, sources and potential human health risk. Environmental Geochemistry and Health 2018c; 40: 791-802.

Cheng C, Zhang L, He Q, et al. 2018. Pollution characteristics of polycyclic aromatic hydrocarbons in park soil in Taiyuan, China. Environmental Chemistry 2018; 37: 2031-2038.

Chung M, Hu R, Cheung K, et al. Pollutants in Hong Kong soils: polycyclic aromatic hydrocarbons. Chemosphere 2007; 67: 464-473.

Dai J, Li S, Zhang Y, et al. Distributions, sources and risk assessment of polycyclic aromatic hydrocarbons (PAHs) in topsoil at Ji’nan city, China. Environmental Monitoring and Assessment 2008; 147: 317-326.

Ding A. Distribution of Polyeyeliearomatie hydroearbon(PAHs) in farmland of Parts of Jiangsu Province and its ecological risk. Nanjing Agricultural University(Doctoral dissertation). 2007.

Ding H, Tao X, KangeLe L, Zhang N. Distribution characteristics and risk analysis of PAHs and PCBs in soils of Lanzhou. Administration and Technique of Environmental Monitoring 2018a; 30: 25-29.

Ding Y, Huang H, Zhang Y, Zheng H, Zeng F, Chen W, et al. Polycyclic aromatic hydrocarbons in agricultural soils from Northwest Fujian, Southeast China: Spatial distribution, source apportionment, and toxicity evaluation. Journal of Geochemical Exploration 2018b; 195: 121-129.

Dong J. Modelling environmental fate and risk assessment of polycyclic aromatic hydrocarbons in the Lanzhou Area. Lanzhou University(Doctoral dissertation). 2010.

Dong Q, Li J, Wu J, et al. Levels, sources and health risk of typical organic pollutants in soils of Jiangxi Province. Chinese Journal of Soil Science 2016; 47: 1475-1484.

Du F. Distribution characteristics, source and ecological risk assessment of polycyclic aromatic hydrocarbons in surface soil of Shanghai. East China Normal University(Doctoral dissertation). 2014.

Duan Y, Tao S, Wang X, et al. Spatial distribution and sources of PAHs in Tianjin’s topsoil. Acta Pedologica Sinica 2005; 42: 942-947.

Fan X, Liu Z, Ma X, et al. Analysis of pollution characteristics and sources of PAHs in farmland soil around Nanchang city. Environmental Monitoring in China 2009; 25: 109-112.

Fang Z, Lin Y, Wang Y. The characteristic distribution of polycyclic aromatic hydrocarbon compounds in topsoil of Qiannan Area in Guizhou. Journal of Sichuan Normal University(Natural Science) 2014; 37: 380-384.

Feng H, Fu X, Zhao Q, et al. Health risk assessment of polycyclic aromatic hydrocarbons in soils of Ningbo Area, China. Journal of Agro-Environment Science 2011a; 30: 1998-2004.

Feng S, Cao Z, Yang Y, et al. Polycyclic aromatic hydrocarbons, heavy metals, and genotoxicity of the suburban soils from Guangzhou, China. Polycyclic Aromatic Compounds 2013b; 33: 501-518.

Feng X, Teng Y, Li J, et al. Residues characteristics and health risk assessment of PAHs in soils. South-to-North Water Diversion and Water Science and Technology 2011a; 9:114-123.

Feng, A, Zhu, Z, Chen, S, et al. Spatial distribution and risk assessment of polycyclic aromatic hydrocarbons in soils of Shouguang City, North China. China Environmental Science 2013b; 33: 1607-1614.

Gao C, Liu D, Guo M. Pollution characteristics and source analysis of polycyclic aromatic hydrocarbons in farmland soils in typical sewage irrigation districts and highways in Liaoning. Environmental Protection and Circular Economy 2016; 36:46-51.

Gao P. Soil contamination characteristic and health risk assessment from different function areas in Taiyuan City. Dissertation of Shanxi University(Doctoral dissertation). 2016.

Gao Y.. Distribution characteristic and pollution assessment of the road sediment along the major traffic in Nanchang urban area. Nanchang University(Doctoral dissertation). 2008.

Ge C, An Q, Dong Y, et al. Distribution of organic pollutants in agricultural soil in Nanjing city. Resources and Environment in the Yangtze Basin 2006; 15: 361-365.

Ge W, Cheng Q, Chai C, et al. Characteristics of pollution and health risk assessment of polycyclic aromatic hydrocarbons in vegetables from Qingdao suburb. Acta Scientiae Circumstantiae 2017a; 37: 4772-4778.

Ge W, Cheng Q, Chai C, et al. Pollution characteristics and source analysis of polycyclic aromatic hydrocarbons in agricultural soils from Shandong. Environmental Science 2017b; 38: 1587-1596.

Ge X, Jiao X, Yuan X, et al. Environmental geochemical migration of polycyclic aromatic hydrocarbons in Xuzhou urban area. Rock and Mineral Analysis 2008a; 27: 409-412.

Ge X, Xie W, Luo S, et al. Distribution and composition of polycyclic aromatic Hydrocarbons in soil of Miyun and Fangshan area, Beijing. Rock and Mineral Analysis 2004; 23: 132-136.

Ge X, Yan J, Jiao X, et al. Environmental geochemistry of polycyclic aromatic hydrocarbons (PAHs) in the urban area of Xuzhou. Geophysical and Geochemical Exploration 2008b; 32: 622-626.

Gu W, Yao H, Bai J, et al. Risk assessment of soil polycyclic aromatic hydrocarbons pollution at a typical informal e-waste dismantling site in Taizhou, Zhejiang. Chinese Journal of Environmental Management 2019; 11: 67-71.

Han X, Li L, Pan G, et al. Pollution characteristics of polycyclic aromatic hydrocarbons in soils from farmland around the domestic refuse dump. Ecology and Environmental Sciences 2009; 18: 1251-1255.

Han Z, Yang Y, Yang H, et al. Concentrations, sources and ecological risks of polycyclic aromatic hydrocarbons in agricultural soils of Fuzhou city. Journal of Subtropical Resources and Environment 2008; 3: 34-41.

Hao R, Peng S, Song Y, et al. Distribution of priority polycyclic aromatic hydrocarbons in soils in Shantou Specific Economic Zone. Ecology and Environment 2004; 13: 323-326.

Hao R, Song Y, Wan H, Peng S. Polycyclic aromatic hydrocarbons and organochlorine pesticides in agricultural soils from typical area of south subtropics, China. Acta Ecologica Sinica 2007; 27: 2021-2029.

Hao R, Wan H, Song Y, et al. Polycyclic aromatic hydrocarbons in agricultural soils of the southern subtropics, China. Pedosphere 2007; 17: 673-680.

He L, Zhang X. PAHs distribution charateristic and source analysis in the typical soil of Xinluo district, Longyan city. Journal of Fujian Institute of Education 2014a; 15: 115-120.

He L. Organic pollution of the soil in Liaoyang vegetables planting and livestocks cultivating regions. Guangzhou Environmental Science 2011; 26: 41-43.

He X, Song X, Pang Y, et al. Distribution, sources, and ecological risk assessment of SVOCs in surface sediments from Guan River Estuary, China. Environmental Monitoring and Assessment 2014b; 80: 52-58.

He X. Study on distribution characteristic, source and risk assessment of OCPs, PAHs and PFCs in typical regions of Hubei, China. Huazhong Agricultural University(Doctoral dissertation). 2015.

Hou D. Distribution and ecological risk evaluation of persisitent toxic substance in two typical areas of Inner Mongolia, China. Inner Mongolia University(Doctoral dissertation). 2014.

Hu G, Chen S, Zhao Y, Sun C, Li J, Wang H. Persistent toxic substances in agricultural soils of Lishui County, Jiangsu Province, China. Bulletin of Environmental Contamination and Toxicology 2009; 82: 48-54.

Hu J, Zhang G, Di L, et al. Distribution characteristics and source analysis of PAHs in topsoil of Guiyang City, South-west China. Chinese Journal of Ecology 2011a; 30: 1982-1987.

Hu J, Zhang G, Lui C. Pilot study of polycyclic aromatic hydrocarbons in surface soils of Guiyang City, People's Republic of China. Bulletin of Environmental Contamination and Toxicology 2006; 76: 80-89.

Hu J. Characterization of polycyclic aromatic hydrocarbons (PAHs) in airborne particles, waters and soils in Guiyang city. The Chinese Academy of Sciences(Doctoral dissertation). 2005.

Hu L, He G, Liu X, et al. Study of polycyclic aromatic hydrocarbons distribution and their origination in the soil of Beihu Area, China. Advanced Materials Research 2011b; 281: 280-285.

Hu T, Zhang J, Ye C, et al. Status, source and health risk assessment of polycyclic aromatic hydrocarbons (PAHs) in soil from the water-level-fluctuation zone of the Three Gorges Reservoir, China. Journal of Geochemical Exploration 2016; 172: 20-28.

Hua D, Wang Q, Xu H, et al. Polycyclic aromatic hydrocarbons and black carbon in surface soil from traffic areas in Wuhu, China. China Environmental Science 2018; 38: 2253-2263.

Hua Z. The influence of soil and petroleum hydrocarbon properties on the efficiency of petroleum contaminated soil remediation by solvent extraction. Tianjin University(Doctoral dissertation). 2013.

Huang H, Xing X, Zhang Z, Qi S, Yang D, et al. Polycyclic aromatic hydrocarbons (PAHs) in multimedia environment of Heshan coal district, Guangxi: distribution, source diagnosis and health risk assessment. Environmental Geochemistry and Health 2016; 38: 1169-1181.

Huang J. Distribution and sbsorption of polycyclic aromatic hydrocarbons in typical forest soil of Guangzhou. Chinese Academy of Forestry(Doctoral dissertation). 2013.

Hui Y, Zheng M, Liu Z. Distribution of polycyclic aromatic hydrocarbons in sediments from Yellow River Estuary and Yangtze River Estuary,China. Acta Scientiae Circumstantiae 2009; 21: 1625-1631.

Jiang M, Xu S, Xu C, et al. Sources and risk assessment of PAHs in surface soil from urban Green Lands:A case of Gulou district, Fuzhou, China. Journal of Subtropical Resources and Environment 2015; 10: 11-16.

Jiang Y, Wang X, Wang, et al. Levels, composition profiles and sources of polycyclic aromatic hydrocarbons in urban soil of Shanghai, China. Chemosphere 2009; 75: 1112-1118.

Jiang Y, Wang X, Wu M, et al. Contamination, source identification, and risk assessment of polycyclic aromatic hydrocarbons in agricultural soil of Shanghai, China. Environmental Monitoring and Assessment 2011; 183: 139-150.

Jiang Y. Preliminary study on composition, distribution and source identification of persistent organic pollutants in soil of Shanghai. Shanghai University (Doctoral dissertation). 2009.

Jiao H, Bian G, Chen X, Wang S, Zhuang X, Bai Z. Distribution, sources, and potential risk of polycyclic aromatic hydrocarbons in soils from an industrial district in Shanxi, China. Environmental Science and Pollution Research 2017; 24: 12243-12260.

Jiao L, Meng W, Zheng B, et al. Distribution of polycyclic aromatic hydrocarbons (PAHs) in different size fractions of sediments from intertidal zone of Bohai Bay, China. China Environmental Science 2010; 30: 1241-1248.

Jiao W, Lu Y, Li J, et al. Identification of sources of elevated concentrations of polycyclic aromatic hydrocarbons in an industrial area in Tianjin, China. Environmental Monitoring and Assessment 2009a; 158: 581.

Jiao W, Lu Y, Wang T, et al. Polycyclic aromatic hydrocarbons in soils around Guanting Reservoir, Beijing, China. Chemistry and Ecology 2009b; 25: 39-48.

Jiao W, Lu Y, Wang T, Li J, Luo W, Shi Y. Characteristics and sources of polycyclic aromatic hydrocarbons in surface soil from chemical industrial areas. Environmental Science 2009c; 30: 1166-1172.

Jiao W, Wang T, Khim J, et al. Polycyclic aromatic hydrocarbons in soils along the coastal and estuarine areas of the northern Bohai and Yellow Seas, China. Environmental Monitoring and Assessment 2013; 185: 8185-8195.

Jin M, Zhou Y, Wan H. Study on polycyclic aromatic hydrocarbons (PAHs) contents and sources in the surface soil of Huizhou City, South China, based on multivariate statistics analysis. Acta Geochimica 2009; 28: 335-339.

Ke C, Gu Y, Liu Q. Polycyclic aromatic hydrocarbons (PAHs) in exposed-Lawn soils from 28 urban parks in the megacity Guangzhou: occurrence, sources, and human health implications. Archives of Environmental Contamination and Toxicology 2017; 72: 496-504.

Ke Y, Qi S, Chen J, et al. Contents, sources and ecological risks of polycyclic aromatic hydrocarbons in Mianzhu-A’ba soil Profile, Sichuan, China. Safety and Environmental Engineering 2013; 20: 79-83.

Kong L, Shi M, Liang J, et al. Concentration and origin of polycyclic aromatic hydrocarbons in the soil of Dagang Oil Field. Environmental Science and Technology 2018; 41: 151-157.

Kong X. Environmental behaviour of persistent organic pollutants in a typical Karst Sinkhole—a case in Dashiwei Tiankeng Group in Guangxi, China. China University of Geosciences(Doctoral dissertation). 2012.

Lan J, Sun Y, Xiao S, Yuan D. Polycyclic aromatic hydrocarbon contamination in a highly vulnerable underground river system in Chongqing, Southwest China. Journal of Geochemical Exploration 2016; 168: 65-71.

Lang Y, Wang N, Gao H, et al. Distribution and risk assessment of polycyclic aromatic hydrocarbons (PAHs) from Liaohe estuarine wetland soils. Environmental Monitoring and Assessment 2012; 184: 5545-5552.

Li J, Lu Y, Jiao W, et al. Polycyclic aromatic hydrocarbons in soils of an industrial area of China: multivariate analyses and geostatistics. Chemistry and Ecology 2010a; 26: 35-48.

Li J, Lu Y, Shi Y, et al. Environmental pollution by persistent toxic substances and health risk in an industrial area of China. Journal of Environmental Sciences 2011a; 23: 1359-1367.

Li J, Lv Y, Jiao W, et al. Concentration and origin of polycyclic aromatic hydrocarbons in surface soil in the Industrial Area of Tianjin, China. Acta Scientiae Circumstantiae 2008a; 28: 2111-2117.

Li J, Song X, Wei J, et al. Pollution characteristics and source apportionment of polycyclic aromatic hydrocarbons in soils of Shenyang North New Area. Environmental Science 2018; 39: 379-388.

Li J, Song, X, Wei, J, Guo, B, Li, Y. Potential risk assessment of polycyclic aromatic hydrocarbons in soils of Shenyang North New Area, China. Journal of Agro-Environment Science 2017; 6: 2462-2470.

Li J, Wu D, Xu Y, et al. Recent distribution and sources of polycyclic aromatic hydrocarbons in surface soils from Yangtze River Delta. Environmental Science 2016; 37: 253-261.

Li J, Xu S, Zhao Z, et al. Polycyclic aromatic hydrocarbons in water, sediment, soil, and plants of the Aojiang River waterway in Wenzhou, China. Journal of Hazardous Materials 2010b; 173: 75-81.

Li J, Yang H, Xie B, et al. Distribution and source diagnosis of polycyclic aromatic hydrocarbons in agricultural soil at the shore of the Dianchi Lake. Environmental Science and Technology 2015; 38: 31-35.

Li Q, Huang T, Shan X, et al. Distribution and sources of polycyclic aromatic hydrocarbon(PAHs) pollutants in soil of Yanhe River Basin, North of Shaanxi province. Journal of Shaanxi Normal University(Natural Science Edition) 2011b; 39: 76-80.

Li Q. Multi-media distribution and risks of PAHs in the typical city of Poyang Lake Ecological Economic Zone. Nanchang University(Doctoral dissertation). 2016.

Li W, Ma X, Ran D, et al. Distribution of PAHs between soils and pine needles collected from typical areas of Xinjiang. CIESC Journal 2011c; 62: 3263-3268.

Li X, Li P, Lin X, et al. Spatial distribution and sources of polycyclic aromatic hydrocarbons (PAHs) in soils from typical oil-sewage irrigation area, Northeast China Environmental Monitoring and Assessment 2008b; 143: 257-265.

Li X, Ma L, Liu X, et al. Polycyclic aromatic hydrocarbon in urban soil from Beijing, China. Journal of Environmental Sciences 2006; 18: 944-950.

Li X, Shi X, Ma J, et al. Contamination and risk assessment of polycyclic aromatic hydrocarbons in farmland soils of Zhejiang Province. Journal of Agro-Environment Science 2019; 38: 1531-1540.

Li X, Zhao T, Liu B, et al. Polycyclic aromatic hydrocarbons (PAHs) in the surface soils of vegetable plots around the Guanting Reservoir. Acta Scientiae Circumstantiae 2010c; 30: 1492-1498.

Li X, Zhao T, Zhang C, Li P, Li S, Zhao L. Source apportionment of polycyclic aromatic hydrocarbons in agricultural soils of Yanqing County in Beijing, China. Environmental Forensics 2013; 14: 324-330

Li X. Distribution and ecological risk of polycyclic aromatic hydrocarbons in crops and famland from parts of Henan Province. Henan Normal University(Doctoral dissertation). 2015a.

Li X. Soil contamination characteristic and assessment in suburb of Taiyuan city. Shanxi University(Doctoral dissertation). 2015b.

Li Y, Fan Y, Cui X. Study on persistent organic pollution of soils in different land use types in some cities of Heilongjiang Province. Inner Mongolia Science Technology and Economy 2011d; 10: 78-80.

Li Y, Gu H, Huang G, et al. Contamination and health risk assessment of PAHs in irrigation district in southeastern suburb of Beijing. Transactions of the Chinese Society for Agricultural Machinery2017; 9: 237-249.

Li Y, Li F, Chen J, et al. The concentrations, distribution and sources of PAHs in agricultural soils and vegetables from Shunde, Guangdong, China. Environmental Monitoring and Assessment 2008c; 139:61-76.

Li Y, Li F, Zhang T, Yang G, Chen J, Wan H. Pollution assessment, distribution and sources of PAHs in agricultural soils of Pearl River Delta-The biggest manufacturing Base in China. Journal of Environmental Science and Health 2007; 42: 1979-1987

Li, T, Li, W, Yuan, C, Tao, S. Distribution of polycyclic aromatic hydrocarbons in surface soils of Yinchuan Plain and surrounding areas. Journal of Agro-Environment Science 2014; 33: 2136-2142.

Liao L. Geochemistry of harmful components in soils of urban and suburban areas ofBaotou, Inner Mongolia. China University of Geosciences (Beijing) (Doctoral dissertation). 2013.

Liao S, Lang Y, Wang Y. Distribution and ecological risk assessment of PAHs in soil from Liaohe Estuarine Westland. Environmental Chemistry 2011; 30: 423-429.

Lin C, Liu J, Wang R, et al. Polycyclic aromatic hydrocarbons in surface soils of kunming, China: concentrations, distribution, sources, and potential risk. Journal of Soil Contamination 2013; 22: 753-766.

Lin C. Distribution and sources of polycyclic aromatic hydrocarbons in urban surface soil of Kunming. Dissertation of Kunming University of Science and Technology(Doctoral dissertation). 2012.

Lin J, Ni J, Yang H, et al. Concentrations, sources and ecological risks of polycyclic aromatic hydrocarbons in the topsoils of Quanzhou city, China. Environmental Science 2011; 32: 2074-2080.

Lin Y, Fang Z, Wang Z, et al. Pollution characteristic of polycyclic aromatic hydrocarbon in topsoil of Guizhou, Qiannan District. Guizhou Agricultural Science 2015; 43: 159-161.

Liu F, Liu Y, Wang J, et al. Pollution characteristics of polycyclic aromatic hydrocarbons in the topsoil of Taiyuan city, north China. Earth Science Frontiers 2008; 15: 155-160.

Liu G, Niu J, Wang L, et al. Contamination and risk assessment of PAHs in agricultural soil from wastewater irrigated area. Environmental Chemistry 2017; 36: 1622-1629.

Liu G, Yu L, Li J, et al. PAHs in soils and estimated air–soil exchange in the Pearl River Delta, South China. Environmental Monitoring and Assessment 2011a; 173: 861-870.

Liu G. Preliminary study on regional geochemistry of polycyclic aromatic hydrocarbons in the Pearl River Delta. The Chinese Academy of Sciences(Doctoral dissertation). 2005.

Liu H, Yu X, Liu Z, Sun Y. Occurrence, characteristics and sources of polycyclic aromatic hydrocarbons in arable soils of Beijing, China. Ecotoxicology and Environmental Safety 2018a; 159: 120-126.

Liu J, Liu G, Zhang J, Yin H, Wang R. Occurrence and risk assessment of polycyclic aromatic hydrocarbons in soil from the Tiefa coal mine district, Liaoning, China. Journal of environmental monitoring 2012; 14: 2634.

Liu J, Liu Y J, Liu Z, et al. Source apportionment of soil PAHs and human health exposure risks quantification from sources: the Yulin National Energy and Chemical Industry Base, China as case study. Environmental Geochemistry and Health 2018b; 206: 1-16.

Liu Q, Liu Y, Hu D, Wang X. Polycyclic aromatic hydrocarbons in traffic soil and Pinus needles of Beijing, China. Chemical Speciation and Bioavailability 2011b; 23: 243-248

Liu S, Xia X, Yang L, et al. Polycyclic aromatic hydrocarbons in urban soils of different land uses in Beijing, China: distribution, sources and their correlation with the city's urbanization history. Journal of Hazardous Materials 2010a; 177: 1085-1092.

Liu W, Liu W, Zhang W, et al. Study on the soil PAHs pollution characteristics of main parks in Shenyang. Journal of Meteorology and Environment 2018c; 34: 69-74.

Liu Z, Teng Y, Huang B, et al. Distribution and sources analysis of PAHs in farmland soils in areas typical of the Yangtze River Delta, China. Acta Pedologica Snica 2010b; 47: 1110-1117.

Long B. Contents and sources of polycyclic aromatic hydrocarbons in vegetable and soil of Nanning City, China. Guangxi University(Doctoral dissertation). 2017.

Long M, Long B, Liang Y, et al. Analysis of contents and sources of polycyclic aromatic hydrocarbons in vegetable production base of Nanning city. China Vegetables 2017; 3: 52-57.

Lu J, Zhao Y, Hao L, et al. Distribution and risk evaluation of polycyclic aromatic hydrocarbons of agricultural soil in the middle Jilin province. Jilin University Journal Social Sciences Edition 2010; 40: 683-688.

Lu M, Yuan D, Ouyang T, et al. Source analysis and health risk assessment of polycyclic aromatic hydrocarbons in the topsoil of Xiamen Island. Journal of Xiamen University (Natural Science) 2008; 47: 451-456.

Luo X, Chen S, Mai B, et al. Distribution, source apportionment, and transport of PAHs in sediments from the Pearl River Delta and the Northern South China Sea. Archives of Environmental Contamination and Toxicology 2008; 55: 11-20.

Lv J, Bi C, Chen Z, et al. Distribution and ecological risk assessment of polycyclic aromatic hydrocarbons in agricultural soil of the Chongming Island in Shanghai. Chin J. Environmental Science 2012; 33: 4270-4275.

Lv J, Shi R, Cai Y, Liu Y. Assessment of polycyclic aromatic hydrocarbons (PAHs) pollution in soil of suburban areas in Tianjin, China. Bulletin of Environmental Contamination and Toxicology 2010; 85: 5-9.

Lv J, Xu R, Zhang Q, et al. Primary investigation of the pollution status of polycyclic aromatic hydrocarbons (PAHs) in water and soil of Xuanwei and Fuyuan, Yunnan Province, China. Chinese Science Bulletin 2009; 54: 3528-3535.

Ma J, Qiu X, Zhou Y, et al. PAHs pollution and spatial distribution in agricultural soils of Dongguan. Acta Scientiarum Naturalium Universitatis Pekinensis 2011; 47: 149-158.

Ma J, Zhou Y. Soil pollution by polycyclic aromatic hydrocarbons: A comparison of two Chinese cities. Journal of Environmental Sciences 2011; 23: 1518-1523.

Ma L, Chu S, Wang X, et al. Polycyclic aromatic hydrocarbons in the surface soils from outskirts of Beijing, China. Chemosphere 2005a; 58: 1355-1363.

Ma L, Chu S, Xu X. Organic contamination in the greenhouse soils from Beijing suburbs, China. Journal of Environmental Monitoring 2003; 5: 786-790.

Ma L, Derek M, Wang X, et al. Simultaneous analysis of organic pollutants in soils by gas chromatography and gas chromatography-mass spectrometry. International Journal of Environmental Analytical Chemistry 2005b; 85: 89-98.

Ma W, Ding X, Long J, et al. Source analysis and risk assessment of polycyclic aromatic hydrocarbons (PAHs) in paddy soils. Soil and Sediment Contamination 2017; 26: 277-293.

Ma W, Li Y. Polycyclic aromatic hydrocarbons and polychlorinated biphenyls in topsoils of Harbin, China. Archives of Environmental Contamination and Toxicology 2009; 57: 670-678.

Ma W, Liu L, Qi H, et al. Polycyclic aromatic hydrocarbons in water, sediment and soil of the Songhua River Basin, China. Environmental Monitoring and Assessment 2013; 185: 8399-8409.

Ma W, Liu L, Tian C, et al. Polycyclic aromatic hydrocarbons in Chinese surface soil: occurrence and distribution. Environmental Science and Pollution Research 2015; 22: 4190-4200.

Ma W. Study on PCBs, PAHs and OCPs in soil in Harbin City. Harbin Institute of Technology(Doctoral dissertation). 2007.

Ma X, Ran Y, Gong J, et al. Concentrations and inventories of polycyclic aromatic hydrocarbons and organochlorine pesticides in watershed soils in the Pearl River Delta, China. Environmental Monitoring and Assessment 2008; 145: 453-464.

Ma X, Ran Y, Xing B, et al. Concentration ofpolycyclic aromatic hydrocarbons in vegetable soils of the Pearl River Delta. Acta Scientiae Circumstantiae 2007; 27: 1727-1733.

Maimaiti S, Palida, Y, Nuerbiya H. Contents and sources of polycyclic aromatic hydrocarbons in soils around Urumqi City. Soils 2016; 48: 1166-1171.

Miao Y, Kong X, Li C. Distribution, sources, and toxicity assessment of polycyclic aromatic hydrocarbons in surface soils of a heavy industrial city, Liuzhou, China. Environmental Monitoring and Assessment 2018; 190: 164-174.

Miao Y, Kong X, Zou S, et al. Environmental geochemical characteristics of PAHs in soils in Nanning city. Safety and Environmental Engineering 2013; 20: 95-101.

Miao Y, Kong X. Distribution characteristics of polycyclic aromatic hydrocarbons in environmental media in Nanning city. Environmental Science 2016; 37: 4333-4340.

Miao Y. Level, source apportionment and ecological risk assessment of polycyclic aromatic hydrocarbons in soil from Shanghai. Shanghai University(Doctoral dissertation). 2013.

Ni H, Qin P, Cao S, et al. Fate estimation of polycyclic aromatic hydrocarbons in soils in a rapid urbanization region, Shenzhen of China. Journal of Environmental Monitoring 2011; 13: 313-318.

Ni J, Chen W, Yang H, et al. Concentrations and sources of soil PAHs in various functional zones of Fuzhou City. China Environmental Science 2012; 32: 921-926.

Ni J, Li X, Guo J , et al. Distribution pattern, sources and potential risks of polycyclic aromatic hydrocarbons in urban soils of Fuzhou City, China. Molecular Environmental Soil Science at the Interfaces in the Earth’s Critical Zone 2010; 01: 226-228.

Pan L, Wang S, Ma J, Fang D. Gridded field observations of polycyclic aromatic hydrocarbons in soils from a typical county in Shanxi Province, China. Archives of Environmental Contamination and Toxicology 2015; 68: 323-329.

Peng C, Chen W, Liao X, et al. Polycyclic aromatic hydrocarbons in urban soils of Beijing: status, sources, distribution and potential risk. Environmental Pollution 2011; 159: 802-808.

Peng C, Ouyang Z, Wang M, et al. Vegetative cover and PAHs accumulation in soils of urban green space. Environmental Pollution 2012a; 161: 36-42.

Peng C, Wang M, Chen W. Spatial Analysis of PAHs in Soils along an Urban–Suburban–Rural Gradient: scale effect, distribution patterns, diffusion and influencing factors. Scientific Reports 2016a; 6: 37185-37194.

Peng C, Wang M, Ouyang Z, Jiao W, Chen W. Characterization and potential risks of polycyclic aromatic hydrocarbons in green space soils of educational areas in Beijing. Chin. Environmental Science 2012b; 33: 592-598.

Peng C, Wang M, Zhao Y, Chen W. Distribution and risks of polycyclic aromatic hydrocarbons in suburban and rural soils of Beijing with various land uses. Environmental Monitoring and Assessment 2016b; 188: 162-173.

Peng C. Distribution and health risk assessment of polycyclic aromatic hydrocarbons in urban soil of Beijing. Hunan Agricultural University(Doctoral dissertation). 2009.

Ping L, Luo Y, Zhang H, et al. Distribution of polycyclic aromatic hydrocarbons in thirty typical soil profiles in the Yangtze River Delta region, east China. Environmental Pollution 2007; 147: 358-365.

Ping X, Lin M, Wang T, et al. Distribution and source of polycyclic aromatic hydrocarbons (PAHs) in top soil of Huangyan district. Journal of Zhejiang Agricultural Sciences 2019; 60: 157-158.

Qiao M, Cai C, Huang Y, et al. Characterization of PAHs contamination in soils from metropolitan region of Northern China. Bulletin of Environmental Contamination and Toxicology 2010; 85: 190-194.

Qiu H, Liu Y, Xie X, et al. Distribution characteristics and source analysis of polycyclic aromatic hydrocarbons in salinized farmland soil from the Oil Mining Area of the Yellow River Delta. Environmental Science 2019; 40: 1-16

Shan D, Liu X, Gan L, et al. Pollution characteristics and health risk assessment of polycyclic aromatic hydrocarbons in agricultural soils in Dongying city, Shandong province. Journal of Northeast Agricultural University 2019; 50: 77-87.

Shao X, Xu Y, Zhang W, et al. Polycyclic Aromatic Hydrocarbons (PAHs) Pollution in Agricultural Soil in Tianjin, China. Journal of Soil Contamination 2015; 24: 343-351.

Shen C, Chen Y, Huang S, et al. Dioxin-like compounds in agricultural soils near e-waste recycling sites from Taizhou area, China: Chemical and bioanalytical characterization. Environment International 2009a; 35: 50-55.

Shen F, Zhu L. Concentration and distribution of PAHs in vegetables grown near an iron and steel industrial area. Environmental Science 2007; 28: 669-672.

Shen J, Wang X, Duo K. Study on the monitoring of organic pollution situation of classic agricultural soil. Journal of Zhengzhou University of Light Industry (Natural Science) 2009b; 24: 20-23+37.

Shi B Zhang B, Wang Y, et al. Source apportionment of polycyclic aromatic hydrocarbons in surface soil from Nanchong. Chemical Research and Application 2010; 22: 835-840.

Shi B, Wu Q, Ouyang H, et al. Characteristics and sources of polycyclic aromatic hydrocarbons in surface soil from industrial areas of Baise, Guangxi. China Environmental Science 2014; 34: 2593-2601.

Shi B, Wu Q, Ouyang H, Liu X, Zhang J, Zuo W. Distribution and source apportionment of polycyclic aromatic hydrocarbons in the surface soil of Baise, China. Environmental Monitoring and Assessment 2015a; 187: 232-245.

Shi R, Xu M, Liu A, et al. Characteristics of PAHs in farmland soil and rainfall runoff in Tianjin, China. Environmental Monitoring and Assessment 2017; 189: 558.

Shi Y, Sun Y, Liang Z, et al. Altitudinal gradient distribution and source analysis of PAHs content of topsoil in Jinfo Mountain, Chongqing. Environmental Science 2015b; 36: 1417-1424.

Song N, Ma J, Yu Y, Yang Z, Li Y. New observations on PAH pollution in old heavy industry cities in northeastern China. Environmental Pollution 2015; 205: 415-423.

Sun J. Studies on contaminative characteristics of persistent toxic substances in surface soils from Zhejiang Province, China. Zhejiang University of Technology(Doctoral dissertation). 2012.

Sun L, Geng Y, Joseph S, et al. Measurement of polycyclic aromatic hydrocarbons (PAHs) in a Chinese brownfield redevelopment site: the case of Shenyang. Ecological Engineering 2013; 53: 115-119.

Sun N, Lu C, Gao X, et al. Distribution and source of polycyclic aromatic hydrocarbons (PAHs) in soil of East Qingzang Plateau. Environmental Science 2007; 28: 664-668.

Sun X, Shi C, Xu S, et al. Concentration and Sources of Polycyclic Aromatic Hydrocarbons in Surface Soil of North Suburban Shanghai, China. Research of Environmental Sciences 2008; 21: 140-144.

Sun Y, Qi S, Li H, et al. Concentrations, sources and health risk assessment of polycyclic aromatic hydrocarbons in soils collected along the banks of Minjiang River, Fujian, China. China China Environmental Science 2016; 36: 1821-1829.

Sun Y, Sun G, Zhou Q, et al. Polycyclic aromatic hydrocarbon (PAH) contamination in the urban topsoils of Shenyang, China. Journal of Soil Contamination 2012; 21: 901-917.

Sun Z, Liu J, Zhuo S, Chen Y, Zhang Y, Shen H, et al. Occurrence and geographic distribution of polycyclic aromatic hydrocarbons in agricultural soils in eastern China. Environmental Science and Pollution Research 2017; 24: 12168-12175.

Tang L, Tang X, Zhu Y, , et al. Distribution of Polycyclic Aromatic Hydrocarbons in soil of Beijing. Journal of PLA University of Science and Technology 2004; 05: 95-99.

Tang L, Tang X, Zhu Y, et al. Contamination of polycyclic aromatic hydrocarbons (PAHs) in urban soils in Beijing, China. Environment International 2005; 31: 822-828.

Tang X, Shen C, Cheema SA, Chen L, Xiao X, Zhang C, et al. Levels and distributions of polycyclic aromatic hydrocarbons in agricultural soils in an emerging e-waste recycling town in Taizhou area, China. Journal of Environmental Science and Health 2010a; 45: 1076-1084.

Tang X, Shen C, Chen L, et al. Inorganic and organic pollution in agricultural soil from an emerging e-waste recycling town in Taizhou area, China. Journal of Soils and Sediments 2010b; 10: 895-906.

Tang X, Shen C, Shi D, et al. Heavy metal and persistent organic compound contamination in soil from Wenling: an emerging e-waste recycling city in Taizhou area, China. Journal of Hazardous Materials 2010c; 173: 653-660.

Tao S, Cui Y, Xu F, et al. Polycyclic aromatic hydrocarbons (PAHs) in agricultural soil and vegetables from Tianjin. Science of the Total Environment 2004; 320: 11-24.

Tao S, Wang W, Liu W, et al. Polycyclic aromatic hydrocarbons and organochlorine pesticides in surface soils from the Qinghai-Tibetan plateau. Journal of Environmental Monitoring 2011; 13: 175-181.

Tian D, Yan W, Kang W, et al. Study on concentration and distribution characteristics of polycyclic aromatic hydrocarbons (PAHs) in soils of cinnamomum camphora stand. Scientia Silvae Sinicae 2006; 42: 12-16.

Wang B. Study on characterizations of organic micro-pollutants and heavy metals in soil at typical industrial district. Lanzhou Jiaotong University(Doctoral dissertation). 2013.

Wang C, Wang X, Gong P, Yao T. Polycyclic aromatic hydrocarbons in surface soil across the Tibetan Plateau: Spatial distribution, source and air-soil exchange. Environmental Pollution 2014a; 184: 138-144.

Wang C, Wu S, Zhou S, Wang H, Li B, Chen H, et al. Polycyclic aromatic hydrocarbons in soils from urban to rural areas in Nanjing: Concentration, source, spatial distribution, and potential human health risk. Science of the Total Environment 2015a; 527-528: 375-383.

Wang D, Luo M, Zhang Q, Dai L, Liu Y, Wang L, Liu X. Distribution characteristics of polycyclic aromatic hydrocarbons in different functional zones of soils from Xiqing District in Tianjin,China. Journal of Agro-Environment Science 2012a; 31: 2374-2380.

Wang J, Cao X, Liao J, et al. Carcinogenic potential of PAHs in oil-contaminated soils from the main oil fields across China. Environmental Science and Pollution Research International 2015b; 22: 10902-10909.

Wang J, Zhang X, Ling W, Liu R, Liu J, Kang F, et al. Contamination and health risk assessment of PAHs in soils and crops in industrial areas of the Yangtze River Delta region, China. Chemosphere 2017; 168: 976-987.

Wang K, Shen Y, Zhang S, et al. Application of spatial analysis and multivariate analysis techniques in distribution and source study of polycyclic aromatic hydrocarbons in the topsoil of Beijing, China. Environmental Geology 2009a; 56: 1041-1050.

Wang L, Xu X, Lu X. Composition, source and potential risk of polycyclic aromatic hydrocarbons (PAHs) in vegetable soil from the suburbs of Xianyang City, Northwest China: a case study. Environmental Earth Sciences 2016; 75: 56-68.

Wang L, Zhang P, Wang L, Zhang W, Shi X, Lu X, et al. Polycyclic aromatic hydrocarbons in urban doil in the demi-arid vity of Xi’an, Northwest China: composition, distribution, sources, and relationships with soil properties. Archives of Environmental Contamination and Toxicology 2018a; 75: 351-366.

Wang L, Zhang S, Wang L, Zhang W, Shi X, Lu X, et al. Concentration and risk evaluation of polycyclic aromatic hydrocarbons in urban doil in the typical semi-arid city of Xi’an in Northwest China. International Journal of Environmental Research and Public Health 2018b; 15: 607-621.

Wang S, Ni H, Sun J, et al. Polycyclic aromatic hydrocarbons in soils from the Tibetan Plateau, China: distribution and influence of environmental factors. Environmental Science-Processes and Impacts 2013; 15: 661-667.

Wang S, Shi F, Pei Z, et al. Evaluation and analysis of farmland soils pollution status of songnen plain: for example Suihua area of Heilongjiang province. Journal of Northeast Agricultural University 2015c; 46: 75-83.

Wang W, Simonich S, Giri B, et al. Spatial distribution and seasonal variation of atmospheric bulk deposition of polycyclic aromatic hydrocarbons in Beijing-Tianjin region, North China. Environmental Pollution 2011; 159: 287-293.

Wang W, Simonich S, Miao X, et al. Concentrations, sources and spatial distribution of polycyclic aromatic hydrocarbons in soils from Beijing, Tianjin and surrounding areas, North China. Environmental Pollution 2010a; 158: 1245-1251.

Wang X, Gao J, Shen J. The pollution distribution characteristics of polycyclic aromatic hydrocarbons in representative region soil. Administration and Technique of Environmental Monitoring 2014b; 2: 22-25.

Wang X, Miao Y, Zhang Y, Li Y, Wu M, Yu G. Polycyclic aromatic hydrocarbons (PAHs) in urban soils of the megacity Shanghai: occurrence, source apportionment and potential human health risk. Science of the Total Environment 2013; 447: 80-89.

Wang X, Zhang P, Zhou H, et al. Polycyclic aromatic hydrocarbons (PAHs) in urban topsoils: concentration and source analysis in Xuzhou, China. International Journal of Environmental Studies 2012b; 69: 602-615.

Wang X, Zheng Y, Liu R, et al. Medium scale spatial structures of polycyclic aromatic hydrocarbons in the topsoil of Tianjin area. Journal of Environmental Science and Health Part B 2003; 38: 327-335.

Wang X, Zuo Q, Duan Y, Liu W, Cao J, Tao S. Factors affecting spatial variation of polycyclic aromatic hydrocarbons in surface soils in North China Plain. Environmental Toxicology and Chemistry 2012c; 31: 2246-2252.

Wang X. The content and distribution of polycyclic aromatic hydrocarbons（PAHs）of urban forest soil in Nanjing. Nanjing Forestry University(Doctoral dissertation). 2015.

Wang Y, Qi S, Chen J, et al. Concentration, distribution and sources of polyaromatic hydrocarbons in soils from the Karst Tiankengs, South China. Bulletin of Environmental Contamination and Toxicology 2009b; 83: 720-726.

Wang Y, Tian Z, Zhu H, et al. Polycyclic aromatic hydrocarbons (PAHs) in soils and vegetation near an e-waste recycling site in South China: concentration, distribution, source, and risk assessment. Science of the Total Environment 2012d; 439: 187-193.

Wang Y, Xue R, Li J, et al. Compositional fractionation of polyaromatic hydrocarbons in the karst soils, South China. Environmental Earth Sciences 2012e; 66: 2013-2019.

Wang Z, Chen J, Qiao X, Yang P, Tian F, Huang L. Distribution and sources of polycyclic aromatic hydrocarbons from urban to rural soils: A case study in Dalian, China. Chemosphere 2007a; 68: 965-971.

Wang Z, Chen J, Tian F, et al. Application of factor analysis with nonnegative constraints for source apportionment of soil polycyclic aromatic hydrocarbons (PAHs) in Liaoning, China. Environmental Forensics 2010b; 11: 161-167.

Wang Z, Chen J, Yang P, Qiao X, Tian F. Polycyclic aromatic hydrocarbons in Dalian soils: distribution and toxicity assessment. Journal of Environmental Monitoring 2007b; 9: 199-204.

Wang Z, Liu Z, Yang Y, et al. Distribution of PAHs in tissues of wetland plants and the surrounding sediments in the Chongming wetland, Shanghai, China. Chemosphere 2012f; 89: 221-227.

Wang Z. Regional study on soil polycyclic aromatic hydrocarbons in Liaoning: patterns, sources and cancer risks. Dalian University of Technology(Doctoral dissertation). 2007.

Wei X. Pollution and potential ecological risk assessment of organochlorine pesticide and heavy metals in farmland soils in Jiaogang Lake Basin. Anhui University(Doctoral dissertation). 2016.

Wei Y, Bao L, Wu C, He Z, Zeng E. Association of soil polycyclic aromatic hydrocarbon levels and anthropogenic impacts in a rapidly urbanizing region: Spatial distribution, soil–air exchange and ecological risk. Science of The Total Environment 2014; 473-474: 676-684.

Wong M, Wu S, Deng W, et al. Export of toxic chemicals - a review of the case of uncontrolled electronic-waste recycling. Environmental Pollution 2007; 149: 131-140.

Wu D, Liu H, Liu M, et al. Pollution characteristics and health risk assessment of polycyclic aromatic hydrocarbons in soil from a typic peri-urban area. Environmental Chemistry 2018; 37: 1565-1574.

Wu D, Wang Y, Liu W, et al. Concentrations and component profiles PAHs in surface soils and wheat grains from the cornfields close to the steel smelting industry in Handan, Hebei province. Environmental . Science 2016; 37, 740-749.

Xiao C, Tai C, Zhao T, et al. Distribution characteristics of polycyclic aromatic hydrocarbons in soil around the Jiaozuo power plant. Acta Scientiae Circumstantiae 2008a; 28: 1579-1585.

Xiao R, Du X, He X, et al. Vertical distribution of polycyclic aromatic hydrocarbons (PAHs) in Hunpu wastewater-irrigated area in northeast China under different land use patterns. Environmental Monitoring and Assessment 2008b; 142:23-34.

Xiao Y, Tong F, Kuang Y, et al. Distribution and source apportionment of polycyclic aromatic hydrocarbons (PAHs) in forest soils from urban to rural areas in the Pearl River Delta of Southern China. International Journal of Environmental Research and Public Health 2014; 11: 2642-2656.

Xie T, Zhang S, Yang R. Contamination levels and source analysis of polycyclic aromatic hydrocarbons and organochlorine pesticides in soils and Grasses from lake catchments in the Tibetan plateau. Environmental Science 2014; 35: 2680-2690.

Xie W, Chen A, Li J, Liu Q, Yang H, Lu Z. County-scale distribution of polycyclic aromatic hydrocarbons in topsoil of the Yellow River Delta Region. Journal of Environmental Science and Health 2012; 47: 1419-1427.

Xing W, Luo Y, Wu L, et al. Spatial distribution of PAHs in a contaminated valley in Southeast China. Environmental Geochemistry and Health 2006; 28: 89-96.

Xing X, Qi S, Zhang J, Wu C, Zhang Y, Yang D, et al. Spatial distribution and source diagnosis of polycyclic aromatic hydrocarbons in soils from Chengdu Economic Region, Sichuan Province, western China. Journal of Geochemical Exploration 2011; 110: 146-154.

Xu F, Qi S, Gao Y, Xing X. Vertical distribution of polycyclic aromatic hydrocarbons in soils of representative spot in Mianyan. Resources and Environment in the Yangtze Basin 2009; 18: 192-196.

Xu P, Tao B, Ye Z, Zhao H, Ren Y, Zhang T, et al. Polycyclic aromatic hydrocarbon concentrations, compositions, sources, and associated carcinogenic risks to humans in farmland soils and riverine sediments from Guiyu, China. Journal of Environmental Sciences 2016; 48: 102-111.

Xu S, Jiang X, Wang L, et al. Polycyclic aromatic hydrocarbons (PAHs) pollutants in sediments of the Yangtze River and the Liaohe River. China Environmental Science 2000; 20: 128-131.

Xu Y, Dai S, Meng K, et al. Occurrence and risk assessment of potentially toxic elements and typical organic pollutants in contaminated rural soils. Science of the Total Environment 2018; 630: 618-629.

Xue J, Zhang J, Huang X. Distributions and origins of polycyclic aromatic hydrocarbons in urban soil profiles at Wuhan. Environ. Environmental Science and Technology 2011; 34: 1-40.

Yang B, Xue N, Zhou L, Li F, Cong X, Han B, et al. Risk assessment and sources of polycyclic aromatic hydrocarbons in agricultural soils of Huanghuai plain, China. Ecotoxicology and Environmental Safety 2012; 84: 304-310.

Yang C. Investigation and analysis on environmental quality and countermeasures of soil in Xingtai City. Hebei Argicultural University(Doctoral dissertation). 2016a.

Yang G, Zhang T, Gao S, Guo Z, Wan H, Gao Y. Source and distribution characteristics of polycyclic aromatic hydrocarbons in agricultural soils in the Pearl River Delta. Environmental Science 2007; 28: 2350-2354.

Yang H, Zhang H, Liu Y, et al. Distribution, assessment and sources analysis of persistent organic toxic chemicals in sediments of Dongting Lake. China Environmental Science 2017; 37: 1530-1539.

Yang J, Yu F, Yu Y, Zhang J, Wang R, Srinivasulu M, et al. Characterization, source apportionment, and risk assessment of polycyclic aromatic hydrocarbons in urban soil of Nanjing, China. Journal of Soils and Sediments 2017; 17: 1116-1125.

Yang R, Zhang S, Li A, et al. Altitudinal and spatial signature of persistent organic pollutants in soil, Lichen, Conifer Needles, and Bark of the Southeast Tibetan Plateau: implications for sources and environmental cycling. Environmental Science and Technology 2013; 47: 12736-12743.

Yang W, Lang Y H, Bai J, et al. Quantitative evaluation of carcinogenic and non-carcinogenic potential for PAHs in coastal wetland soils of China. Ecological Engineering 2015a; 74: 117-124.

Yang W, Lang Y, Li G. Cancer risk of polycyclic aromatic hydrocarbons (PAHs) in the soils from Jiaozhou Bay wetland. Chemosphere 2014; 112: 289-295.

Yang X, Li S, Li L, et al. Preliminary study on the composition characteristics of polycyclic aromatic hydrocarbons in surface soils of industrial sites and traffic sites in Guangzhou. Acta Scientiarum Naturalium Universitatis Sunyatseni 2008; 47: 93-97.

Yang X, Ren D, Sun W, Li X, Huang B, Chen R, et al. Polycyclic aromatic hydrocarbons associated with total suspended particles and surface soils in Kunming, China: distribution, possible sources, and cancer risks. Environmental Science and Pollution Research 2015b; 22: 6696-6712

Yang X. Pollution characteristic of polycyclic aromatic hydrocarbon in atmospheric particlulate matters and surface soil of Kunming City. Kunming University of Science and Technology(Doctoral dissertation). 2015.

Yang Y. The distribution and source identification of PAHs in the sediments and soils around Poyang Lake. Nanchang University(Doctoral dissertation). 2016b.

Ye L. Distribution and sources of PAHs in surface soils in different functional areas of Shanxi Province. Dissertation of Xi’an University of Architecture and Technology(Doctoral dissertation). 2013.

Yin C, Jiang X, Yang X, et al. Polycyclic aromatic hydrocarbons in soils in the vicinity of Nanjing, China. Chemosphere 2008; 73: 389-394.

Ying L, Ling C, Zhao J F, et al. Polycyclic aromatic hydrocarbons in the surface soil of Shanghai, China: concentrations, distribution and sources. Organic Geochemistry 2010; 41: 355-362.

Yu G, Zhang Z, Yang G, Zheng W, Xu L, Cai Z. Polycyclic aromatic hydrocarbons in urban soils of Hangzhou: status, distribution, sources, and potential risk. Environmental Monitoring and Assessment 2014; 186: 2775-2784.

Yu L, Li J, Liu G,et al. Polycyclic aromatic hydrocarbons in surface soils of the Pearl River Delta, south China. Ecology and Environment 2007; 16: 1683-1687.

Yuan G, Qin J, Li J, et al. Persistent organic pollutants in soil near the Changwengluozha glacier of the Central Tibetan Plateau, China: their sorption to clays and implication. Science of the Total Environment 2014a; 472:309-315.

Yuan G, Wu H, Fu S, et al. Persistent organic pollutants (POPs) in the topsoil of typical urban renewal area in Beijing, China: Status, sources and potential risk. Journal of Geochemical Exploration 2014b; 138: 94-103.

Yuan G, Wu L, Sun Y, Li J, Li J, Wang G. Polycyclic aromatic hydrocarbons in soils of the central Tibetan Plateau, China: Distribution, sources, transport and contribution in global cycling. Environmental Pollution 2015a; 203: 137-144.

Yuan H, Li T, Ding X, Zhao G, Ye S. Distribution, sources and potential toxicological significance of polycyclic aromatic hydrocarbons (PAHs) in surface soils of the Yellow River Delta, China. Marine Pollution Bulletin 2014c; 83: 258-264.

Yuan J, Wang X, Zhou J, et al. Distribution, source and risk analysis of polycyclic aromatic hydrocarbons in top-soil from Jinan City. Environmental Chemistry 2015b; 34: 166-171.

Yuan Z, Liu G, Da C, Wang J, Liu H. Occurrence, sources, and potential toxicity of polycyclic aromatic hydrocarbons in surface soils from the Yellow River Delta Natural Reserve, China. Archives of Environmental Contamination and Toxicology 2015c; 68: 330-341.

Yuan, H, Zhao, G, Pang, S, et al. Polycyclic aromatic hydrocarbons (PAHs) exposure and their source analysis in the northern wetland of the Yellow River Delta. Marine Geology and Quaternary Geology 2008; 28: 57-62.

Zeng X, Li F, Zhou K, et al. Distribution of polycyclic aromatic hydrocarbons (PAHs) in the sediments and mangrove plants at Baguang wetland, Shenzhen. Environmental Science and Technology 2013; 36: 368-373.

Zhang D, Cao S, Sun J, et al. Occurrence and spatial differentiation of polycyclic aromatic hydrocarbons in surface soils from Shenzhen, China. Environmental Science 2014a; 35: 711-718.

Zhang D, Liu N, Ye Q. Distribution, sources and potential risk assessment of PAHs in surface soil of Qingdao City. Urban Environment and Urban Ecology 2016a; 29: 7-12.

Zhang D, Wang J, Zeng H. Soil Polycyclic aromatic hydrocarbons across urban density zones in Shenzhen, China: occurrences, source apportionments, and spatial risk assessment. Pedosphere 2016b; 26: 676-686.

Zhang G. Study on the distribution characteristics of polycyclic aromatic hydrocarbons in agricultural soils in Zhuzhou city. Environmental Monitoring in China 2009a; 25: 51-54.

Zhang H, Luo Y, Huang M, et al. Hong Kong soil researches Ⅲ. PAHs contents in soils and their origins Acta Pedologica Sinica 2005b; 42: 936-941.

Zhang H, Luo Y, Wong M, et al. Distributions and concentrations of PAHs in Hong Kong soils. Environmental Pollution 2006; 141: 107-114.

Zhang J, Fan S, Du X, et al. Accumulation, allocation, and risk assessment of polycyclic aromatic hydrocarbons (PAHs) in soil-brassica chinensis system. Plos One 2015; 10: e0115863.

Zhang J, Wu J, Liu Y. Polycyclic aromatic hydrocarbons in urban green spaces of Beijing: concentration, spatial distribution and risk assessment. Environmental Monitoring and Assessment 2016c; 188: 511-521.

Zhang J, Yu F, Yang J, et al. Accumulation of polycyclic aromatic hydrocarbons and their correlation with black carbon in urban forest soil of Nanjing City, China. Journal of Nanjing Forestry University (Natural Sciences Edition) 2018; 42: 75-80.

Zhang Q, Shi Y, Dong L, et al. Investigation on PAHs pollution in soil of Huaian typical area. Administration and Technique of Environmental Monitoring 2007; 19: 22-24.

Zhang R, Zhang X, He L. Distribution and source of polycyclic aromatic hydrocarbons (PAHs) in soils of XinLuo District, Longyan City. Applied Mechanics and Materials 2011a; 71-78: 2907-2911.

Zhang T, Wan H, Yang G, et al. Distribution of polycyclic aromatic hydrocarbons in agricultural soil and vegetables of Foshan City in the Pearl River Delta. Acta Scientiae Circumstantiae 2008a; 28: 2375-2384.

Zhang T, Wang H, Zhou J, Yang G, Gao Y. Distribution and source of polycyclic aromatic hydrocarbons (PAHs) in topsoil of Shenzhen. Ecology and Environmental Sciences 2008b; 17: 1032-1036.

Zhang T, Yang G, Wan F, et al. Concentration, indicators, and origin of polycyclic aromatic hydrocarbons in the surface soil in Dongguan. Soils 2005b; 37: 265-271.

Zhang W, Wang H, Zhang R, et al. Bacterial communities in PAH contaminated soils at an electronic-waste processing center in China. Ecotoxicology 2010; 19: 96-104.

Zhang X, Cui Y, Zhang G, et al. Distribution characteristics of polycyclic aromatic hydrocarbons in agricultural soils in Taiyuan basin. Journal of Taiyuan University of Science and Technology 2014b; 35: 231-235.

Zhang X, Yang J, Liu M, et al. Distribution characteristics and source analysis of PAHs in farmland soils along Shanghai traffic artery. China Environmental Science 2019; 39: 741-749.

Zhang X, Zhang F. Pollution characteristics and ecological risk assessment of polycyclic aromatic hydrocarbons in agricultural soils of Hohhot, China. Agricultural Science and Technology 2017; 18: 747-752.

Zhang Y, Wang J. Distribution and source of polycyclic aromatic hydrocarbons (PAHs) in the surface soil along main transportation routes in Jiaxing City, China. Environmental Monitoring and Assessment 2011b; 182: 535-543.

Zhang Y. Distribution, sources and content of PAHs in soils of Tibet. Taiyuan University of Sciene and Technology(Doctoral dissertation). 2012.

Zhang Z, Huang J, Yu G, et al. Occurrence of PAHs, PCBs and organochlorine pesticides in the Tonghui River of Beijing, China. Environmental Pollution 2004; 130: 249-261.

Zhang Z, Wang X, Ha L, et al. The source and distribution characteristics of polycyclic aromatic hydrocarbons in surface sediments of Xiamen Western Bay. Marine Science Bulletin 2001; 20: 35-39.

Zhang M, Teng M, et al. Distribution characteristics of polycyclic aromatic hydrocarbons in top soils in Zunyi, Guizhou province. Journal of Jiangxi Normal University 2009b; 33: 716-720.

Zhao H, Zhao J, Xu Y, et al. Spatial distribution, sources and risk assessment of polycyclic aromatic hydrocarbons in a rapid urbanization city: Shenzhen. Journal of Ecology and Rural Environment 2019; 35: 38-45.

Zhao J, Zhou H, Lu J, et al. Distribution and source of polycyclic aromatic hydrocarbons (PAHs) in soils of Baiyang-dian Area. Chinese Journal of Ecology 2009; 28: 901-906.

Zhao L, Hou H, Shangguan Y, Cheng B, Xu Y, Zhao R, et al. Occurrence, sources, and potential human health risks of polycyclic aromatic hydrocarbons in agricultural soils of the coal production area surrounding Xinzhou, China. Ecotoxicology and Environmental Safety 2014; 108: 120-128.

Zhao W. Pollution and risk analysis of heavy metals and polycyclic aromatic hydrocarbons in urban soils of Kaifeng City. Henan University(Doctoral dissertation). 2017.

Zhao X. Study on distribution behavior characteristic of PAHs in the forest soil and plant leaves. Yanbian University(Doctoral dissertation). 2016.

Zheng H, Xing X, Hu T, et al. Biomass burning contributed most to the human cancer risk exposed to the soil-bound PAHs from Chengdu Economic Region, western China. Ecotoxicology and Environmental Safety 2018; 159: 63-70.

Zheng T, Ran Y, Chen L. Polycyclic aromatic hydrocarbons (PAHs) in rural soils of Dongjiang River Basin: occurrence, source apportionment, and potential human health risk. Journal of Soils and Sediments 2014a; 14: 110-120.

Zheng T, Ran Y, Chen L. Polycyclic aromatic hydrocarbons in the rural soils of Dongjiang River Basin: distribution and human health risks. Ecology and Environmental Sciences 2014b; 23: 657-661

Zhou B, Zhang C, Jiang J, et al. Characteristics and sources of PAHs in top-soil of Xi’an city. Environmental Science and Technology 2012a; 35: 97-87.

Zhou L, Xue N, Li F, et al. Distribution, source analysis and risk assessment of polycyclic aromatic hydrocarbons in farmland soils in Huanghuai Plain. China Environmental Science 2012b; 32: 1250-1256.

Zhou W, Li J, Hu J, et al. Distribution, sources, and ecological risk assessment of polycyclic aromatic hydrocarbons (PAHs) in soils of the central and eastern areas of the Qinghai Tibetan plateau. Environmental Science 2018a; 39: 1413-1420.

Zhou Y, Lu X. Assessment of pollution, sources and risks of polycyclic aromatic hydrocarbons in soil from urban parks in Xi’an City, China. Environmental Science 2017; 38: 4800-4808.

Zhou Y, Lu X. Sources Identification and risk assessment of polycyclic aromatic hydrocarbons in surface soils of educational areas in Xi’an. Earth and Environment 2018b; 46: 381-387.

Zhou Y. Study on soil heavy metals and polycyclic aromatic hydrocarbons pollution in different functional areas of Xi’an City. Shaanxi Normal University(Doctoral dissertation). 2018.

Zhu H. The content, composition and their distribution of polycyclic aromatic hydrocarbons in the soil-plant system of an electronic waste pollution area in Qingyuan, Guangdong. Nanjing Agricultural University(Doctoral dissertation). 2013.

Zhu L, Chen Y, Zhou R. Distribution of polycyclic aromatic hydrocarbons in water, sediment and soil in drinking water resource of Zhejiang Province, China. Journal of Hazardous Materials 2008; 150: 308-316.

Zhu Y, Tian J, Wei E, et al. Characteristics, sources apportionment and ecological risks assessment of polycyclic aromatic hydrocarbons in soils of Tianjin, China. Environmental Chemistry 2014; 33: 248-255.

Zhuang W, Wang X, Yao W, et al. Distribution and sources of polycyclic aromatic hydrocarbons in the surface sediments of Quanzhou Bay, China. Environmental Chemistry 2011; 30: 928-934.

Zou Z, Tang H, Liu Y. Source and distribution characteristics of polycyclic aromatic hydrocarbons in agricultural soils in Beijing suburbs. Environmental Chemistry 2013; 32: 874-880.

Zuo Q, Duan Y, Yang Y, et al. Source apportionment of polycyclic aromatic hydrocarbons in surface soil in Tianjin, China. Environmental Pollution 2007; 147: 303-310.

#### **Table 1.** PCA-MLR model of PAHs data for surface soil samples

| **PAHs** | **Urban** | | | **Suburban** | | **Rural** | | **IMIA** | |
| --- | --- | --- | --- | --- | --- | --- | --- | --- | --- |
|  | **PC1** | **PC2** | **PC3** | **PC1** | **PC2** | **PC1** | **PC2** | **PC1** | **PC2** |
| Naphthalene (Nap) | **.768** | -.409 | -.056 | .678 | -.255 | **.914** | -.266 | **.869** | .058 |
| Acenaphthylene (Acy) | .586 | -.485 | -.225 | .609 | .050 | **.715** | -.306 | .613 | **.731** |
| Acenaphthene (Ace) | **.728** | -.125 | -.188 | .797 | .125 | .548 | -.204 | **.876** | .183 |
| Fluorine (Flu) | **.878** | -.247 | .158 | **.847** | -.381 | **.913** | -.159 | **.960** | .237 |
| Phenanthrene (Phe) | .492 | -.101 | **.839** | .637 | -.453 | **.941** | -.194 | .687 | .695 |
| Anthracene (Ant) | .167 | -.178 | -.183 | **.767** | -.002 | **.918** | -.276 | **.922** | -.180 |
| Fluoranthene (Flt) | .699 | .559 | .012 | **.925** | -.106 | .332 | **.783** | **.818** | -.011 |
| Pyrene (Pyr) | **.895** | .012 | .367 | **.865** | -.067 | .463 | .542 | **.936** | -.264 |
| Benza(a)anthracene (BaA) | **.928** | .075 | -.171 | **.929** | -.033 | **.944** | -.229 | **.849** | -.003 |
| Chrysene (Chr) | **.885** | .002 | .357 | **.805** | -.254 | **.970** | -.115 | **.895** | -.183 |
| Benzo(b)fluoranthene (BbF) | **.825** | .226 | -.170 | **.887** | .226 | .342 | **.866** | .499 | -.129 |
| Benzo(k)fluoranthene (BkF) | **.911** | .060 | -.159 | **.895** | -.156 | **.950** | -.222 | .633 | .545 |
| Benzo(a)pyrene (BaP) | **.941** | .091 | -.159 | **.928** | -.037 | **.904** | .291 | **.882** | -.379 |
| Dibenz(a,h)anthracene (DahA) | .142 | **.716** | .018 | .480 | .654 | **.797** | .078 | .636 | -.421 |
| Indeno(1,2,3-cd)pyrene (IcdP) | **.937** | .086 | -.147 | .557 | **.706** | .508 | .503 | **.939** | -.245 |
| Benzo(g,h,i)perylene (BghiP) | **.939** | .002 | -.142 | .453 | .692 | .440 | .693 | **.884** | -.252 |
| Variance contribution rate(%) | 60.094 | 8.890 | 7.897 | 59.284 | 12.480 | 58.048 | 18.384 | 66.895 | 12.525 |
| Cumulative(%) | 60.094 | 68.984 | 76.881 | 59.284 | 71.764 | 58.048 | 76.432 | 66.895 | 79.420 |
| Contribution of PAHs sources(%) | 65.639 | 17.747 | 16.614 | 76.831 | 23.169 | 91.002 | 8.998 | 65.843 | 34.157 |

Notes: The bold data indicate compounds with loading coefficients higher than 0.700.

#### **Table 2.** Pearson correlation coefficients for PAHs and energy indicators

| **item** | **LMW** | **HMW** | **Provincial PAHs accumulation** | **Petroleun reserves** | **Natural gas reserves** | **Coal reserves** | **ΣP-coal** | **ΣP&C-crude oil and coal** |
| --- | --- | --- | --- | --- | --- | --- | --- | --- |
| LMW | 1 |  |  |  |  |  |  |  |
| HMW | -0.023 (N=31) | 1 |  |  |  |  |  |  |
| Provincial PAHs accumulation | 0.410* (N=31) | 0.521** (N=31) | 1 |  |  |  |  |  |
| Petroleun reserves | 0.695** (N=22) | 0.155 (N=22) | 0.476* (N=22) | 1 |  |  |  |  |
| Natural gas reserves | 0.101 (N=24) | 0.571** (N=24) | 0.643** (N=24) | 0.327 (N=24) | 1 |  |  |  |
| Coal reserves | -0.009 (N=30) | 0.655** (N=30) | 0.491** (N=30) | 0.234 (N=22) | 0.226 (N=24) | 1 |  |  |
| ΣP-coal | -0.048 (N=30) | 0.677** (N=30) | 0.540** (N=30) | 0.190 (N=21) | 0.281 (N=23) | 0.956** (N=29) | 1 |  |
| ΣP&C-crude oil and coal | -0.006 (N=30) | 0.565** (N=30) | 0.492** (N=30) | 0.276 (N=21) | 0.187 (N=23) | 0.873** (N=29) | 0.940** (N=30) | 1 |

Note：ΣP-coal means Total production of coal from 1997 to 2016 in China; ΣP&C-crude oil and coal means total production and consumption of crude oil and coal from 1997 to 2016 in China; * means significant correlation at 0.05 level; ** means significant correlation at 0.01 level.

Detailed data information of this table’s data can be found in supplementary information.

| Province | LMW  /(µg/kg) | HMW  /(µg/kg) | Provincial PAHs accumulation  /(µg/kg) | Petroleun reserves(2017)  /(10^4^ tons) | Natural gas reserves(2017)  /(10^4^ tons) | Coal reserves(2017)  /(10^4^ tons) | ΣP-coal（1997-2016）/(10^4^ tons) | ΣP&C-crude oil and coal（1997-2016）/(10^4^ tons) |
| --- | --- | --- | --- | --- | --- | --- | --- | --- |
| Anhui | 138.12 | 1141.04 | 268334.13 | 238.50 | 0.25 | 82.37 | 190523.66 | 405897.61 |
| Beijing | 731.79 | 1234.94 | 43762.53 |  |  | 2.66 | 13444.01 | 80304.21 |
| Chongqing | 419.83 | 381.85 | 57425.60 | 266.90 | 2726.90 | 18.03 | 65018.46 | 155124.73 |
| Fujian | 117.58 | 391.51 | 166785.16 |  |  | 3.98 | 30379.57 | 150337.83 |
| Gansu | 4436.93 | 618.51 | 678600.50 | 28261.70 | 318.03 | 27.32 | 70198.68 | 176442.89 |
| Guangdong | 133.71 | 268.88 | 136127.56 | 16.40 | 0.59 | 0.23 | 3926.71 | 306561.42 |
| Guangxi | 495.36 | 413.10 | 463823.94 | 154.00 | 1.58 | 0.90 | 13571.45 | 106488.97 |
| Guizhou | 103.19 | 464.70 | 192333.84 |  | 6.10 | 110.93 | 228317.39 | 412495.28 |
| Hebei | 1410.36 | 206.39 | 487659.34 | 26576.40 | 338.03 | 43.27 | 156043.13 | 621184.56 |
| Heilongjiang | 2863.46 | 416.38 | 1136955.88 | 42665.80 | 1302.33 | 62.28 | 158033.73 | 486776.09 |
| Henan | 110.80 | 112.74 | 145021.66 | 4427.00 | 74.77 | 85.58 | 302942.41 | 690560.14 |
| Hong Kong | 57.06 | 192.42 | 816.46 | 452.30 | 24.35 | 1.19 |  |  |
| Hubei | 1034.92 | 578.39 | 240435.91 | 1185.90 | 46.87 | 3.20 | 19602.44 | 228214.59 |
| Hunan | 243.63 | 859.68 | 324141.13 |  |  | 6.62 | 103670.88 | 282627.87 |
| Inner Mogilia | 328.62 | 2834.42 | 2209243.50 | 8381.30 | 9630.49 | 510.27 | 936492.86 | 1333944.97 |
| Jiangsu | 297.18 | 698.53 | 227517.78 | 2729.50 | 23.31 | 10.39 | 47063.24 | 445346.55 |
| Jiangxi | 123.68 | 1851.15 | 224741.78 |  |  | 3.36 | 47787.38 | 152648.91 |
| Jilin | 656.45 | 1603.38 | 524345.06 | 17500.60 | 731.25 | 9.71 | 61048.51 | 236634.46 |
| Liaoning | 514.94 | 869.42 | 449303.75 | 14351.60 | 154.54 | 26.73 | 117121.80 | 493280.47 |
| Ningxia | 68.80 | 42.60 | 85675.68 | 2432.40 | 274.44 | 37.45 | 89752.93 | 179538.35 |
| Qinghai | 60.64 | 17.19 | 482828.97 | 8252.30 | 1354.44 | 12.39 | 20523.10 | 49371.24 |
| Shaanxi | 444.89 | 1821.37 | 517335.09 | 38375.60 | 7802.50 | 162.93 | 484155.93 | 743274.71 |
| Shandong | 767.58 | 284.33 | 284332.19 | 29412.20 | 334.93 | 75.67 | 265283.74 | 915517.36 |
| Shanghai | 241.74 | 993.57 | 15553.44 |  |  |  |  | 126325.63 |
| Shanxi | 456.83 | 2404.84 | 642011.38 |  | 413.75 | 916.19 | 1157484.34 | 1675970.80 |
| Sichuan | 370.33 | 1581.71 | 739014.81 | 623.40 | 13191.61 | 53.21 | 137809.10 | 314115.19 |
| Tianjin | 666.32 | 592.15 | 33484.87 | 3349.90 | 274.91 | 2.97 | 0.00 | 144480.15 |
| Tibet | 77.70 | 16.41 | 165920.78 |  |  | 0.12 | 15.42 | 15.42 |
| Xinjiang | 2782.33 | 979.42 | 1498492.00 | 59576.30 | 10251.78 | 162.31 | 145979.24 | 372304.39 |
| Yunnan | 97.75 | 285.19 | 684715.50 | 12.20 | 0.47 | 59.58 | 111198.24 | 241165.24 |
| Zhejiang | 175.19 | 338.54 | 143766.45 |  |  | 0.43 | 846.71 | 245598.92 |

Notes: Data about amounts of energy reserves (petroleum, gas and coal) in the Chinese provincial administrative areas, and the production and consumption of coal and petroleum in 1997 -2016 were obtained from the public service platform of Chinese energy data in 2016 (Energy information association) (http://www.eia.org.cn).

#### **Table 3.** Comparison of PAHs concentration (µg/kg) observed in this study with those found in other region of the world in different soil types.

|  | China | USA^a^ | India^b^ | Turkey^c^ | UK^d^ | Korea^e^ |
| --- | --- | --- | --- | --- | --- | --- |
| Urban | 1479.97  (0.02-39891) | 2732  (362-16462) | 5524.25  (1550.9-11460) | 2243.5 | 68000 | 390  (65-1200) |
| Suburban | 523.34  (3.51-6248) | 172  (70-448) | - | 55.9 | 41000 | 236  (23.3-2834) |
| Rural | 293.95  (0.38-5910) | 170  (78.7-497) | 1458  (827-2095) | 152 | 590 | 220  (92-450) |
| IMIA | 8800.77  (5.1-97680) | - | 3920  (1190- 12700) | 4628 | - | 2515 |

Notes: Detailed information about references a, b, c, d and e of this table can be found in supplementary information.

**References:**

a:

Yuan, S., Li, K., Chen, T., Bi, X., Wang, Q., 2014. Soil contamination by polycyclic aromatic hydrocarbons at natural recreational areas in Delaware, USA. Environ Earth Sci. 72, 387-398. https://doi.org/10.1007/s12665-013-2959-x.

b:

Agarwal, T., Khillare, P.S., Shridhar, V., Ray, S., 2009. Pattern, sources and toxic potential of PAHs in the agricultural soils of Delhi, India. J Hazard Mater. 163, 1033-1039. https://doi.org/10.1016/j.jhazmat.2008.07.058.

Deka, J., Sarma, K,P., Hoque, R,R., 2016. Source contributions of polycyclic aromatic hydrocarbons in soils around oilfield in the Brahmaputra valley. Ecotox Environ Safe. 133, 281-289. https://doi.org/10.1016/j.ecoenv.2016.07.031.

Singh, D.P., Gadi, R., Mandal, T.K., 2012. Levels, sources, and toxic potential of polycyclic aromatic hydrocarbons in urban soil of Delhi, India. Hum Ecol Risk Assess. 18, 393-411. https://doi.org/10.1080/10807039.2012.652461.

c:

Bozlaker, A., Muezzinoglu, A., Odabasi, M., 2008. Atmospheric concentrations, dry deposition and air–soil exchange of polycyclic aromatic hydrocarbons (PAHs) in an industrial region in Turkey. J Hazard Mater. 153, 1093-1102. https://doi.org/10.1016/j.jhazmat.2007.09.064.

Demircioglu, E., Sofuoglu, A., Odabasi, M., 2011. Particle-phase dry deposition and air-soil gas exchange of polycyclic aromatic hydrocarbons (PAHs) in Izmir, Turkey. J Hazard Mater. 186, 328-335. https://doi.org/10.1016/j.jhazmat.2010.11.005.

Karaca, G., 2016. Spatial Distribution of Polycyclic Aromatic Hydrocarbon (PAH) Concentrations in Soils from Bursa, Turkey. Arch Environ Con Tox. 70, 406-417. https://doi.org/10.1007/s00244-015-0248-2.

d:

Vane, C.H., Kim, A.W., Beriro, D.J., Cave, M.R., Knights, K., Moss-Hayes, V., Nathanail, P.C., 2014. Polycyclic aromatic hydrocarbons (PAH) and polychlorinated biphenyls (PCB) in urban soils of Greater London, UK. Appl Geochem. 51, 303-314. https://doi.org/10.1016/j.apgeochem.2014.09.013.

Zohair, A., Salim, A., Soyibo, A.A., Beck, A.J., 2006. Residues of polycyclic aromatic hydrocarbons (PAHs), polychlorinated biphenyls (PCBs) and organochlorine pesticides in organically-farmed vegetables. Chemosphere. 63, 541-553. https://doi.org/10.1016/j.chemosphere.2005.09.012.

e:

Kim, J., Shim, S., Lee. C., 2005. Degradation of phenanthrene by bacterial strains isolated from soil in oil refinery fields in Korea. J Microbiol Biotechn. 15, 337-345. https://doi.org/10.1007/s10295-005-0222-5.

Kwon, H., Choi, S., 2014. Polycyclic aromatic hydrocarbons (PAHs) in soils from a multi-industrial city, South Korea. Sci Total Environ. 470-471, 1494-1501. https://doi.org/10.1016/j.scitotenv.2013.08.031.

Nam, J.J., Song, B.H., Eom, K.C., Lee, S.H., Smith, A., 2003. Distribution of polycyclic aromatic hydrocarbons in agricultural soils in South Korea. Chemosphere. 50, 1281-1289. https://doi.org/10.1016/S0045-6535(02)00764-6.
